# Atherogenesis and iron: from epidemiology to cellular level

**DOI:** 10.3389/fphar.2014.00094

**Published:** 2014-05-05

**Authors:** Francesca Vinchi, Martina U. Muckenthaler, Milene C. Da Silva, György Balla, József Balla, Viktória Jeney

**Affiliations:** ^1^Department of Pediatric Oncology, Hematology and Immunology, University of HeidelbergHeidelberg, Germany; ^2^Molecular Medicine and Partnership Unit, University of HeidelbergHeidelberg, Germany; ^3^MTA-DE Vascular Biology, Thrombosis and Hemostasis Research Group, Hungarian Academy of SciencesDebrecen, Hungary; ^4^Department of Pediatrics, University of DebrecenDebrecen, Hungary; ^5^Department of Medicine, University of DebrecenDebrecen, Hungary

**Keywords:** atherosclerosis, iron, hemoglobin, heme, intraplaque hemorrhage, LDL, oxidative stress, macrophages

## Abstract

Iron accumulates in human atherosclerotic lesions but whether it is a cause or simply a downstream consequence of the atheroma formation has been an open question for decades. According to the so called “iron hypothesis,” iron is believed to be detrimental for the cardiovascular system, thus promoting atherosclerosis development and progression. Iron, in its catalytically active form, can participate in the generation of reactive oxygen species and induce lipid-peroxidation, triggering endothelial activation, smooth muscle cell proliferation and macrophage activation; all of these processes are considered to be proatherogenic. On the other hand, the observation that hemochromatotic patients, affected by life-long iron overload, do not show any increased incidence of atherosclerosis is perceived as the most convincing evidence against the “iron hypothesis.” Epidemiological studies and data from animal models provided conflicting evidences about the role of iron in atherogenesis. Therefore, more careful studies are needed in which issues like the source and the compartmentalization of iron will be addressed. This review article summarizes what we have learnt about iron and atherosclerosis from epidemiological studies, animal models and cellular systems and highlights the rather contributory than innocent role of iron in atherogenesis.

## Introduction

### A role for iron in atherosclerosis: the “iron hypothesis”

The correlation between iron and heart disease was initially proposed by Sullivan in 1981. According to his “iron hypothesis,” iron overload promotes cardiovascular disease, while on the contrary, sustained iron depletion/deficiency exerts a primary protective effect against ischemic heart disease. This theory, continually debated for more than 30 years, was based on the observation that male gender is associated with higher risk of cardiovascular disease, but that the protective effect in women is diminished after menopause (Sullivan, 1981, 1989). Based on results from the Framingham study (Kannel et al., 1976), Sullivan first hypothesized that the regular menstrual loss of iron, rather than the effect of estrogen, protects women against coronary heart disease (CHD). The failure of postmenopausal estrogen replacement to prevent coronary events further supported the iron hypothesis and its link to gender differences in atherosclerosis (Hulley et al., 1998).

The presence of redox-active iron, as well as high expression levels of H- and L-ferritin in human atherosclerotic lesions provided indirect support for the iron hypothesis (Smith et al., 1992; Pang et al., 1996). L-ferritin levels are increased in coronary arteries from patients with coronary artery disease (CAD), indicating that iron accumulates in atherosclerotic plaques (You et al., 2003). Additionally, cholesterol levels in lesions correlate with iron deposits (Stadler et al., 2004). Within the plaque, iron deposition and ferritin induction may be observed in endothelial cells and macrophages in early human lesions, and additionally in vascular smooth muscle cells (VSMCs) in late lesions.

**Figure 1:**
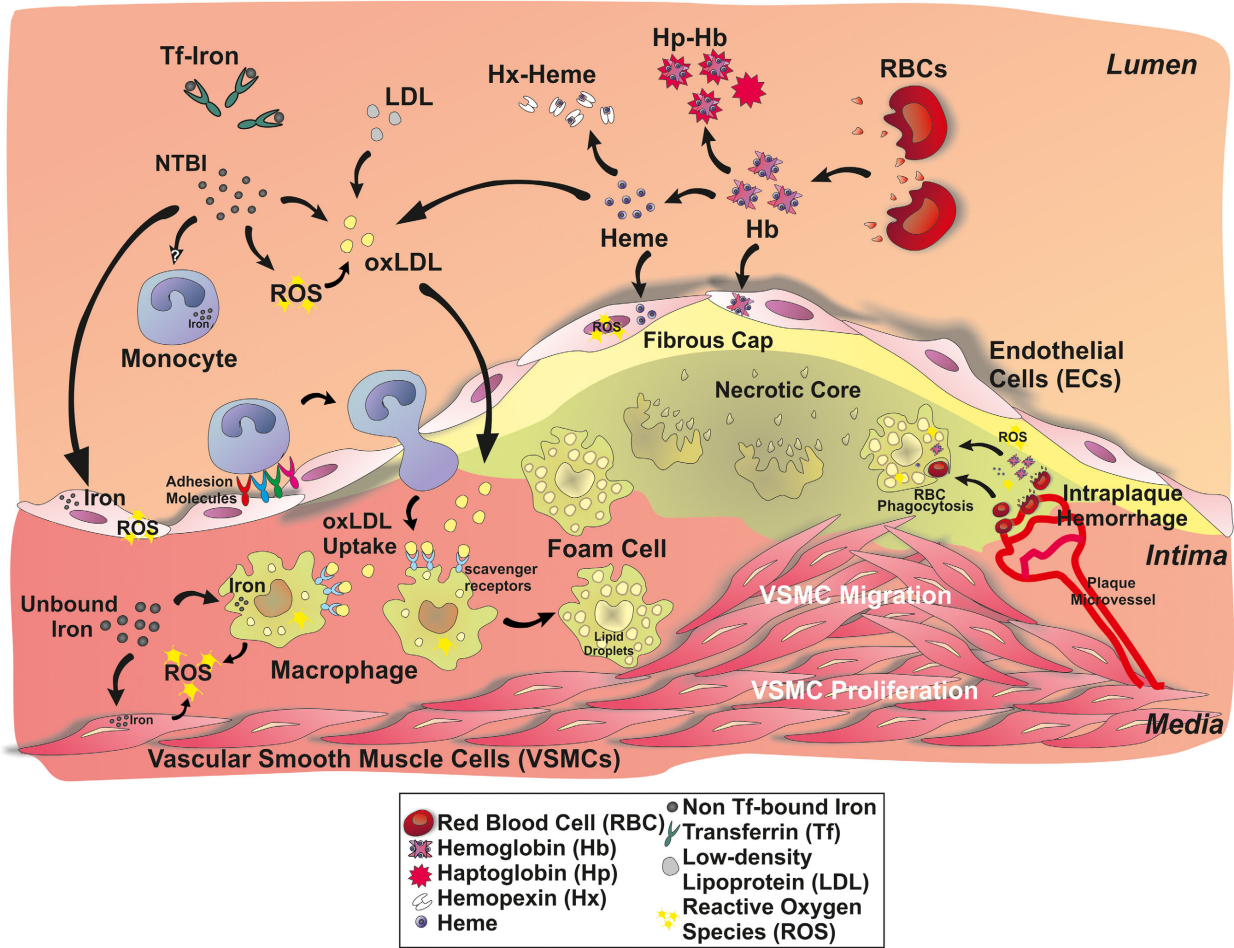
**A role for iron in atherosclerotic lesions**. Iron accumulates in the plaque either as inorganic or hemoglobin-bound iron. Inorganic iron in part derives from circulating transferrin (Tf)-bound iron and non-transferrin-bound iron (NTBI). NTBI is generated in chronic iron overload conditions, such as in iron-loading anemia, hereditary hemochromatosis or secondary hemochromatosis due to blood transfusions. Circulating NTBI is accessible to many cell types in the atherosclerotic plaque: endothelial cells, monocytes/macrophages, and vascular smooth muscle cells (VSMCs). Hemoglobin- and heme-derived iron can access the plaque upon intravascular hemolysis and intraplaque hemorrhage, affecting endothelial cells and macrophages. Hemoglobin (Hb), heme and iron promote endothelial activation, by enhancing adhesion molecule expression. As a consequence, monocyte recruitment is expected to be increased. Circulating iron and Hb oxidize LDLs, thus enhancing subendothelial LDL retention and macrophage progression to foam cells. Iron also affects VSMC proliferation and migration into the lesion, favoring plaque progression.

Iron accumulates in human atherosclerotic lesions (Sullivan, 2009) via different mechanisms. Under normal conditions iron circulates in the bloodstream bound to its carrier protein transferrin (Tf). However, non-transferrin bound iron (NTBI) may be generated during chronic iron overload disorders such as sickle cell disease, thalassemia and transfusional iron overload (Brissot et al., 2012). NTBI is thought to be easily accessible to many cell types within the plaque, likely accumulating in endothelial cells, macrophages, and VSMCs (Figure 1).

Iron can enter into the atherosclerotic lesion in the form of free hemoglobin (Hb), that is released upon intravascular hemolysis or intraplaque hemorrhage (Kolodgie et al., 2003). Intraplaque hemorrhage originates from leaky neovessels invading the intima from the vasa vasorum as the intima thicken, and contributes significantly to the enlargement of the necrotic core (Sakakura et al., 2013). Increasing evidence indicates that plaque neovascularization and vasa vasorum density accompanied by intraplaque hemorrhage is a strong marker for plaque vulnerability (Moreno et al., 2004; Carlier et al., 2005; Virmani et al., 2005; Michel et al., 2011; Sakakura et al., 2013). Following intraplaque hemorrhage, red cells can be taken up by macrophages or they burst extracellularly releasing free Hb. Hb is prone to oxidation, especially in the highly oxidative milieu of the atheroma leading to the formation of metHb and higher oxidation states such as ferrylHb, which can release heme (Figure 1). Altogether, Hb oxidation products, heme and iron exert pro-oxidant and pro-inflammatory effects targeting different cellular (i.e., endothelial cells, smooth muscle cells, macrophages) and acellular components (i.e., low-density lipoprotein, LDL) of the atherosclerotic vessel wall (Figure 1).

The finding that human atherosclerotic plaques contain redox-active iron, that could promote free radical formation and lipid peroxidation, further suggested a role for iron in atherosclerosis that may be eventually responsible for progressive oxidative damage in atherosclerotic lesions. This review article summarizes our current knowledge about the role of Hb, heme, and iron in atherosclerosis by discussing the results of epidemiological studies, and observations in animal models and cellular experiments.

## Altered iron homeostasis and atherosclerosis: epidemiological studies, human cases

### Correlation between markers of iron stores and development of CAD

To evaluate whether iron accumulation in the atherosclerotic plaque is a cause, rather than a consequence, of cardiovascular disease, several epidemiological and perspective studies were conducted since the nineties and many are still ongoing.

The results of several human studies strongly suggested a relationship between body iron levels and atherosclerosis. According to these epidemiological studies, high systemic iron levels, monitored by serum ferritin levels or transferrin saturation, positively correlated with increased risk of myocardial infarction (Salonen et al., 1992; Morrison et al., 1994; Tuomainen et al., 1998; Holay et al., 2012) cardiovascular disease (Rajapurkar et al., 2011), peripheral arterial disease (PAD) (Menke et al., 2009), and mortality rates (Lauffer, 1990). This association was stronger in men with high serum LDL levels, suggesting a synergistic role of high iron and high LDL levels (Salonen et al., 1992; Morrison et al., 1994). Finally, a clear proatherogenic role for iron was suggested by the observation that a 10 mg/L increase in serum ferritin level raised the probability of having at least two atherosclerotic plaques by 3% (Ahluwalia et al., 2010).

Body iron stores correlated with asymptomatic carotid atherosclerosis in healthy men (Syrovatka et al., 2011), an association becomes even more evident in symptomatic atherosclerosis. Plaques from symptomatic patients showed higher iron concentrations, signs of cap rupture and increased cap macrophage activity compared with asymptomatic plaques (Gustafsson et al., 2013). This suggests that the presence of iron in carotid plaques positively correlates with plaque vulnerability for rupture.

Additionally, the description of serum ferritin levels as a risk indicator of carotid lesion progression highlights a clear association between atherosclerosis progression and iron stores (Kiechl et al., 1997).

In agreement with this, serum iron levels directly correlate with cardiovascular disease severity. Serum iron levels were significantly higher in patients with severe atherosclerosis compared to those showing normal, mild, and moderate sings of CADs, thus further strengthening the hypothesis that high iron levels could affect atherosclerosis severity (Bagheri et al., 2013). Collectively, these epidemiological studies clearly identified high body iron levels as a risk factor for atherosclerosis and cardiovascular diseases.

Serum ferritin levels are frequently used to assess body iron status but increasing evidence suggests that this parameter additionally serves as a more general marker of inflammation (Kalantar-Zadeh et al., 2004; Manousou et al., 2011). Thus, some studies evaluated the relationships between serum ferritin, inflammatory cytokines and cardiovascular disease (Haidari et al., 2001; Depalma et al., 2010). Ferritin levels positively correlated with IL-6 and C-reactive protein (hsCRP) levels and were higher in patients that died of acute myocardial infarction vs. survivors, further supporting a rationale for measurement of ferritin levels in patients with atherosclerosis.

### Blood donation and the risk of CAD

The incidence of atherosclerosis in premenopausal women was less than half of that observed in men of the same age (Kiechl and Willeit, 1999). The sex difference disappeared within 5 years after menopause, likely due to increased body iron stores. According to these observations, in 1991 Sullivan proposed that blood donation could prevent cardiovascular disease (Sullivan, 1991). Several studies confirmed the cardiovascular protective effect of blood donation. Blood donation was positively associated with a reduced risk of cardiovascular disease, in particular in non-smoking men with high serum LDL levels (Meyers et al., 1997, 2002; Tuomainen et al., 1997; Salonen et al., 1998). This result was in agreement with the iron hypothesis, according to which the protective effect of blood donation would be more pronounced in men that have a higher body iron load than women. A first randomized clinical trial (FeAST) showed that phlebotomy resulted in clinical benefits and reduction of death in young patients affected by PAD (Sullivan and Katz, 2007; Zacharski et al., 2007). High-frequency blood donation was associated with reduced body iron stores and improved vascular function and blood pressure, reduced oxidative stress, improved markers of cardiovascular risk in blood donors (Zheng et al., 2005; Houschyar et al., 2012). These findings are complemented by the observation that endothelial dysfunction is attenuated by iron chelation in patient with CAD (Duffy et al., 2001). Altogether these findings suggest that iron depletion, by blood donation or iron chelation, significantly lowers the risk of cardiovascular disease, thus supporting the iron hypothesis.

### Association of iron overload and CAD in hemochromatosis

If the iron hypothesis is correct, individuals with iron overload would be expected to show an increased risk and incidence of cardiovascular diseases, thus being optimal study model to test the validity of the hypothesis.

An interesting observation comes from the study of American blacks that compared to American whites and Hispanics are well known for higher ferritin levels throughout their entire life, likely explained by nutritional and genetic factors rather than increased iron intake (Zacharski et al., 2000). Interestingly, the incidence of CHD is higher in African–American than in white men and women (Gillum et al., 1997; Sacco et al., 1998), suggesting an association between body iron and cardiovascular disease.

Hereditary hemochromatosis (HH) is a genetic disorder associated with progressive iron overload, resulting in oxidative stress and organ failure. HH is more common among individuals of Northern European descent and is caused by inherited mutations in proteins implicated in iron transport and regulation, such as the upstream regulators of hepcidin, the human hemochromatosis protein (HFE), hemojuvelin, transferrin receptor (TfR)-2, as well as hepcidin and ferroportin (FPN) (Hentze et al., 2010).

Hemochromatotic patients show vascular dysfunction and increased expression of adhesion molecules that positively correlates to iron overload and NTBI levels (Gaenzer et al., 2002; Kartikasari et al., 2006; Van Tits et al., 2007). These patients further show functional and structural alterations in midsize muscle arteries. In particular, arterial wall thickness is increased before the onset of cardiovascular complications, suggesting that this is an early abnormality in HH. This alteration is reverted by phlebotomy-induced iron depletion, which can also improve the endothelium-dependent vasodilation and the initial radial artery wall stiffening associated with HH (Failla et al., 2000; Gaenzer et al., 2002).

Different cohort studies reported a significantly greater risk of myocardial infarction, cerebrovascular mortality and cardiovascular mortality in carriers of the HFE mutation (Cys282Tyr) (Roest et al., 1999; Tuomainen et al., 1999; Rasmussen et al., 2001). Additionally, patients with genetic hemochromatosis have significant eccentric hypertrophy of the radial artery, although not showing arterial hypertension or evidence of cardiovascular disease.

In contrast to the above reported studies, others failed to find an association between hemochromatosis and the presence or frequency of atherosclerosis and did not succeed in establishing a link between body iron stores and cardiovascular diseases in human populations (Miller and Hutchins, 1994; Sullivan and Zacharski, 2001; Munoz-Bravo et al., 2013). The disagreement among epidemiological studies may result from variations in the validity and reliability of the indicators of iron status. Additionally, the magnitude of the relative risk associated with iron overload might be small, thus the association being obscured by stronger risk factors. Further prospective and experimental studies are needed to confirm the association between the iron status and atherosclerosis.

### The “refined iron hypothesis”: a protective role for iron-depleted macrophages in atherosclerosis

Controversial results from epidemiological studies investigating different types of atherosclerotic events and using various markers for body iron levels present a confusing picture regarding the iron hypothesis. In addition, several studies failed to observe an increased risk or incidence of cardiovascular events in hemochromatotic patients, thus further increasing the confusion concerning an eventual association between iron overload and atherosclerosis (reviewed in Munoz-Bravo et al., 2013). Finally, the description of a potentially protective effect of hemochromatosis against atherosclerosis and cardiovascular diseases was perceived as a “paradox” and considered as clear evidence against the iron hypothesis (Miller and Hutchins, 1994; Sullivan and Zacharski, 2001; Munoz-Bravo et al., 2013). On the basis of these observations Sullivan presented a refinement of his “iron hypothesis” (Sullivan and Zacharski, 2001).

**Figure 2:**
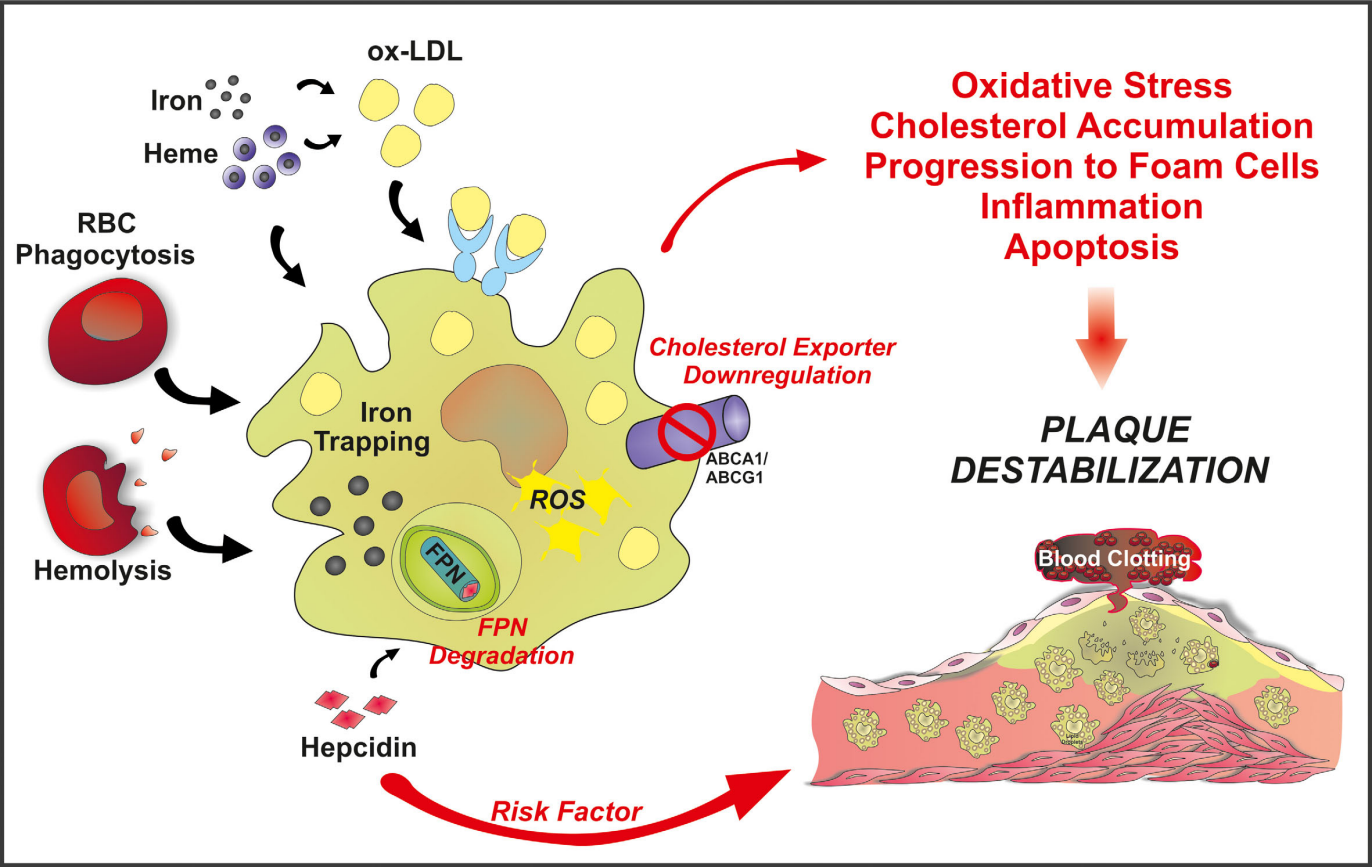
**Schematic overview of the “refined iron hypothesis”: a role for macrophage-retained iron in atherosclerosis**. Iron can accumulate in macrophages as inorganic iron and Hb-iron, upon erytrophagocytosis or hemolysis. Once stored in the cell, iron can be made available to the bloodstream via FPN-mediated export. According to the refined iron hypothesis, high hepcidin levels are considered a risk factor for plaque progression and destabilization. Hepcidin is known to bind to FPN, thus promoting its degradation and blocking iron export. This increases intracellular ROS levels and decreases cholesterol efflux. As a result, the oxidative status alters and LDL accumulation occurs, promoting foam cell formation, inflammation and eventually plaque instability.

Since then the peptide hormone hepcidin has been identified as the master regulator of iron homeostasis. Hepcidin inhibits iron export by binding to FPN and promoting its degradation. By inhibiting FPN, hepcidin prevents iron release from enterocytes into the bloodstream and decreasing iron release from macrophages, thereby reducing the amount of iron systemically available. HH is hallmarked by low levels of hepcidin and/or increased expression of the iron exporter FPN. Therefore, in hemochromatotic patients the FPN-hepcidin circuitry is impaired, leading to increased duodenal iron absorption and reduced iron retention in macrophages (Hentze et al., 2010; Ganz and Nemeth, 2011). Considering the key role of the macrophages in atherogenesis, the selective iron depletion in this cell type was proposed as a mechanism of protection against foam cell formation and atherosclerotic lesion progression (Figure 2). According to this view, the hypothesis postulated by Sullivan that iron depletion protects against atherosclerosis may apply even to hemochromatotic individuals.

According to this view, hepcidin levels may act as a potential iron-dependent risk factor for atherosclerosis by regulating macrophage iron accumulation and atherosclerotic plaque progression (Figure 2). Recently, hepcidin was suggested as a predictor of carotid atherosclerosis. Serum ferritin was found to associate with vascular damage, common carotid thickness and presence of carotid plaques in all patients but not those showing a reduction in hepcidin levels due to heterozygous HFE mutations (Valenti et al., 2011b). Additionally, hepcidin levels and macrophage iron positively correlate with the release of IL-6 and macrophage chemoattractant protein 1 (MCP-1), and vascular damage in high-risk individuals (Valenti et al., 2011a). Collectively, these findings suggest an involvement of iron-loaded macrophages in inflammation and vascular alterations. On the other hand, monocytes from hemochromatotic patients showed reduced ability to accumulate iron and reduced upregulation of MCP-1 and IL-6 (Valenti et al., 2011a). The anti-inflammatory properties of iron-depleted macrophages may help to explain the lack of increased incidence of atherosclerosis in hemochromatotic patients.

Anyway, a direct and definitive demonstration of the refined iron hypothesis in human is still lacking and further studies are needed to fully elucidate the impact of macrophage-stored iron, as well as circulating iron and tissue-stored iron on human lesion formation and progression.

### Thalassemias and sickle cell anemia

β-thalassemia and sickle cell anemia are hereditary blood disorders characterized by anomalies in the synthesis of the β-globin chains of Hb. β-thalassemic and sickle patients show increased plasma iron turnover, iron absorption and tissue iron deposition. Additionally, they have frequent hemolytic events that lead to the release of Hb and heme into the circulation, further increasing the amount of redox-active iron available for the production of reactive oxygen species and lipid peroxidation (Livrea et al., 1998; Brizzi et al., 2002). The release of Hb upon hemolytic events and the enhanced absorption of iron, to support inappropriate erythropoiesis, contribute to the pathogenesis of vasculopathy, a well-known predisposing factor for cardiovascular diseases. Moreover, these patients, usually presenting with severe anemia, require regular red blood cell transfusions (Vichinsky, 2005), further exacerbating iron overload and iron-driven oxidative stress (McLeod et al., 2009).

Iron-dependent peroxidative tissue injury results in arterial stiffness and dysfunction, frequently occurring in thalassemic patients (Kremastinos et al., 1999; Hahalis et al., 2008). Iron overload in patients with beta-thalassemia major lead to alterations in the arterial structures and in the thickness of the carotid arteries (Cheung et al., 2002; Tantawy et al., 2009). Moreover, carotid thickness positively correlated with age, Hb, ferritin and cholesterol levels in these patients (Cheung et al., 2006; Tantawy et al., 2009). As a result, CAD is a quite common cardiovascular complication in thalassemia (Ramakrishna et al., 2003; Ferrara and Taylor, 2005; Aessopos et al., 2007). Patients on a regular transfusion regimen progressively develop clinical manifestations of iron overload associated with heart dysfunction and left ventricular failure (Borgna-Pignatti et al., 2004). Interestingly, iron chelation therapy in thalassemia patients improves arterial function and stiffness (Cheung et al., 2008).

Ischemic complications are the major causes of morbidity and mortality in patients with sickle cell disease (Platt et al., 1994; Switzer et al., 2006). Ischemic events in these patients have been attributed to the effects of Hb polymerization, resulting in sickled cells trapped in the microcirculation (Francis and Johnson, 1991). Nevertheless, different factors other than red blood cell sickling, could contribute to these events, atherosclerosis being one of this.

SCD is an uncommon risk factor for atherosclerosis. However, in the last decades, together with the increased life expectancy of SCD patients, the risk to develop atherosclerosis is significantly increasing. Endothelial dysfunction, hyperhomocysteinemia and activation of platelets are the most likely mechanisms for the development of atherosclerosis in SCD patients (Elsharawy et al., 2009). The presence of excessive circulating Hb, heme, and iron in SCD could have in principle a crucial role in atherosclerosis development, even though a clear experimental proof of this is still missing. Conversely, a paradoxical protective effect of SCD on atherosclerosis and thrombosis was observed in ApoE-null mice transplanted with bone marrow from mice carrying the sickle cell mutation. This effect was abolished by inhibition of HO-1, suggesting that this protection relies on the activity of this enzyme, whose induction is sustained in SCD, due to the high circulating Hb and heme levels (Wang et al., 2013). These observations have the limit that mice were analyzed after 23–28 weeks from bone marrow transplantation, giving an idea of the onset of atherosclerosis but not of the late phases of the disease, in which HO-1 activity could be eventually overwhelmed, thus promoting atherosclerosis progression.

The most common sites of atherosclerosis in these patients are represented by large cerebral arteries (Rothman et al., 1986). Approximately 75% of strokes in sickle cell disease are the result of occlusion of cerebral vessels (Moran et al., 1998). Also pulmonary and splenic arteries are common sites of atherosclerosis in sickle cell disease. One-third of the sickle patients had histological evidence of medial hypertrophy and intimal proliferation in these arteries (de Chadarevian et al., 2001; Graham et al., 2007).

Therefore, thalassemia and sickle cell anemia patients are considered at high atherogenic risk in view of the perturbation of the Hb/heme/iron metabolism that predisposes these patients to oxidative status alterations (Belcher et al., 1999; Switzer et al., 2006).

### Haptoglobin polymorphism and CAD

Extracellular Hb may exert proatherogenic effects via different mechanisms. Free Hb scavenges nitric oxide, an important vasodilator and signaling molecule (reviewed in Rother et al., 2005). Moreover, oxidized Hb species trigger pro-oxidant (reviewed in Balla et al., 2005) and pro-inflammatory effects on vascular endothelium (Silva et al., 2009), and cause lipid-peroxidation (Jeney et al., 2002; Potor et al., 2013).

Efficient mechanisms have evolved to remove extracellular Hb from the circulation to limit its deleterious effects. Haptoglobin (Hp) is an acute-phase plasma protein with the primary function to capture cell-free Hb and chaperon it to macrophages for degradation (reviewed in Alayash, 2011). Hp binding facilitates the removal of Hb from circulation via endocytosis through the CD163 macrophage scavenger receptor (Kristiansen et al., 2001).

The Hp gene is polymorphic in humans, whereby the two functional alleles (hp1 and hp2) can form three genotypes: Hp1-1, Hp2-1, and Hp2-2 with heterogeneous protein structure and functional differences (reviewed in Goldenstein et al., 2012). Differences between antioxidant properties of Hp1-1 and Hp2-2 were examined. An early study showed that Hp1-1 protein is more potent in inhibiting the oxidative actions of extracorpuscular Hb (Melamed-Frank et al., 2001). Contradictory, a recent study described no differences between the two phenotypes in protecting against Hb-driven toxicity (Lipiski et al., 2013). When applied *in vivo* following Hb injection both Hp1-1 and Hp2-2 attenuate Hb-induced blood pressure response with equal efficacy, restrict trans-endothelial diffusion of extracellular Hb equally, and prevent Hb redistribution and renal iron deposition in the same way (Lipiski et al., 2013). Both phenotypes show similar abilities to stabilize the ferryl Hb state, to restrict heme release from the complex, and to prevent Hb-driven LDL oxidation *in vitro* (Lipiski et al., 2013). Immunomodulatory effects of the two phenotypes were compared as well. The Hp1-1-Hb complex induces more robust anti-inflammatory macrophage signaling, leading to the secretion of anti-inflammatory cytokines than that of Hp2-2-Hb complex (Philippidis et al., 2004; Landis et al., 2013).

The Hp polymorphism was investigated as a possible genetic determinant in cardiovascular disease. These epidemiologic studies revealed that the Hp2-2 genotype is a risk factor for cardiovascular complications in both type I and type II diabetic patients (reviewed in Costacou and Levy, 2012). In particular, the Hp2-2 genotype is associated with elevated amounts of iron in atherosclerotic carotid plaques, accompanied by increased levels of oxidation-specific epitopes, increased macrophage infiltration and decreased VSMCs, all events promoting plaque instability (Lioupis et al., 2011, 2012; Purushothaman et al., 2012). In addition, the Hp2-2 genotype is associated with increased circulating oxLDL levels when compared to Hp1-1 or Hp2-1 genotypes (Brouwers et al., 2004). A correlation between the Hp2-2 genotype, carotid plaque instability and increased risk of major cardiovascular diseases was recently described (Ijas et al., 2013).

Collectively, these findings suggest that detoxification of extracellular Hb by Hp acts in an atheroprotective manner. In addition, the Hp2-2 genotype represents a non-modifiable risk factor for cardiovascular diseases. Because Hp1-1 and Hp2-2 inhibit the oxidative actions of extracorpuscular Hb equally, therefore disease association is most probably explained by other functions or properties of the Hp molecule.

### Heme oxygenase-1 (HO-1) and cardiovascular disease

Heme oxygenases catabolize heme to equimolar amounts of biliverdin, carbon monoxide, and free iron, followed by the conversion of biliverdin into bilirubin by biliverdin reductase (Singleton and Laster, 1965; Tenhunen et al., 1968). HO-1 is a stress-inducible isoform of heme oxygenases, encoded by the hmox-1 gene which possesses antioxidant, anti-apoptotic and anti-inflammatory properties (reviewed in Gozzelino et al., 2010; Durante, 2011). These protective mechanisms partially rely on the ability of HO-1 to extract iron from heme. The released iron induces the expression of ferritin, the 24-subunit complex of heavy (H) and light (L) chains, with enormous iron-storage capacity (Eisenstein et al., 1991; Harrison and Arosio, 1996). In addition, both bilirubin and CO, the other two end products of heme degradation exhibit direct anti-oxidant and anti-inflammatory activities (Gozzelino et al., 2010).

An important, but somewhat neglected function of HO-1 is its role in iron recycling (Poss and Tonegawa, 1997). Erythrophagocytosis, subsequent HO-1-mediated heme degradation and iron release from macrophages is a major mechanism in iron recycling, accounting for about 90% of total body iron turnover (reviewed in Hentze et al., 2010).

Accumulating evidences suggest the protective role of HO-1 in atherosclerotic vascular disease (reviewed in Chan et al., 2011). Both the antioxidant bilirubin and the vasodilator CO may contribute to this atheroprotective effect (Siow et al., 1999; Mayer, 2000; Parfenova et al., 2012; Erkan et al., 2013). Low bilirubin levels are associated with endothelial dysfunction and increased intima-media thickness (Erdogan et al., 2006), whereas high plasma bilirubin concentrations are linked to low incidence of cardiovascular disease (Schwertner et al., 1994) and stroke (Kimm et al., 2009). Differences in plasma bilirubin levels may arise from the variation of HO-1 activity in humans.

In the human hmox-1 promoter a GT repeat microsatellite polymorphism exists, leading to higher hmox-1 transcriptional activity and subsequently higher HO-1 expression in individuals having shorter GT repeats compared to subjects with longer GT repeats. A number of studies investigated the relationship between this gene polymorphism and the risk of cardiovascular disease, with conflicting results. Some studies revealed that shorter GT repeats in the hmox-1 promoter region are associated with lower incidence and/or progression of CAD (Kaneda et al., 2002; Liang et al., 2013), whereas others argue against a relevant role of this polymorphism in cardiovascular diseases (Lublinghoff et al., 2009).

Progressive atherosclerotic lesion destabilization with subsequent plaque rupture is a key event predisposing to acute thrombus formation and coronary artery occlusion (Schwartz et al., 2007). Autopsy studies reveal that the risk of plaque rupture mainly depends on the composition of the plaque rather than its size (Kolodgie et al., 2004). Severe macrophage infiltration, a necrotic core and a thin fibrous cap are the main characteristics of vulnerable plaques (Kolodgie et al., 2004). In humans, HO-1 expression is increased in atherosclerotic lesions and closely correlates with plaque instability and pro-inflammatory markers. The observation that HO-1 induction reverses plaque progression from a vulnerable plaque to a more stable phenotype suggests that HO-1 expression may act as a compensatory atheroprotective mechanism (Cheng et al., 2009).

By contrast, HO-1 deficiency in humans leads to severe vascular pathologies (Yachie et al., 1999). A 6-year old boy with inactivating mutations of the HO-1 gene presented with severe intravascular hemolysis associated with persistent endothelial damage. Autopsy examination revealed the presence of aortic fatty streaks and fibrous plaques at this young age, highlighting the atheroprotective function of HO-1 (Yachie et al., 1999). More recently, another case of HO-1 deficiency in a young girl was reported, with evidence of severe endothelial damage, as suggested by raised inflammatory markers, von Willebrand factor and coagulopathy (Radhakrishnan et al., 2011). Since free circulating heme promotes endothelial damage, the lack of functional HO-1 likely results in a form of vasculitis or endothelial injury syndrome. This may therefore increase their susceptibility to develop atherosclerosis.

Taken together, these findings prove a crucial role for HO-1 in the maintenance of vascular homeostasis and counteraction of atherosclerosis.

## Altered iron homeostasis and atherosclerosis: animal models

### Iron overload and iron deficiency in atherosclerosis

The effect of iron in atherogenesis was tested using different hypercholesterolemic animal models. In an initial study that intramuscular administration of iron dextrane augmented the formation of atherosclerotic lesions in hypercholesterolemic rabbits (Araujo et al., 1995). In contrast, another group using the same rabbit model described that iron dextrane injection significantly decreased lesion formation by about 50% by reducing plasma cholesterol levels (Dabbagh et al., 1997). Kirk et al. observed a reduction (about 50%) in plaque area in apoE deficient mice fed with a 2% carbonyl iron containing standard diet in spite of that dietary iron overload caused a modest (30%) rise in plasma triglyceride and cholesterol levels (Kirk et al., 2001).

Other studies took the opposite approach and examined the effect of iron restriction on atherogenesis. In this regard, atherosclerotic lesions in mice fed a low-iron diet were significantly smaller than those found in control littermates (Lee et al., 1999). Reduced plaque size in the low-iron group was associated with lower levels of circulating autoantibodies to oxLDL, and the diminished occurrence of thiobarbituric acid reactive epitopes in the lesions (Lee et al., 1999). This was explained by the observation that dietary iron restriction increases plaque stability via elevated collagen and reduces matrix metalloproteinase-9 expressions in the lesion (Lee et al., 2003). Consistently, iron chelation by DFO lowers the iron content of the lesions and inhibits atherosclerotic lesion development in cholesterol-fed rabbit (Minqin et al., 2005) as well as in apoE deficient mice (Zhang et al., 2010). Other than an effect on atherosclerosis, several studies showed that iron depletion by chelation significantly reduces endothelial activation and vascular dysfunction in animal models (Ishizaka et al., 2005). Recently, a combined therapy of iron chelator and antioxidant was observed to restore iron-induced brain vascular dysfunction in rats (Sripetchwandee et al., 2014), supporting the idea that iron promotes earlier steps in atherogenesis.

### Hemochromatosis models

Although there is support for the idea that iron is detrimental for atherosclerosis, the validity of the original iron hypothesis has not been tested in models of genetic iron overload, such as hemochromatotic mice. To date, several mouse models of hemochromatosis are available, such as HFE-null, Hamp-null, HJV-null, and BMP6-null mice (Fleming et al., 2011) but atherosclerosis progression has not been assessed in any of them. Future studies will have to dissect the contribution of systemic iron overload and macrophage iron deficiency in hemochromatotic mouse models for atherosclerosis in order to better understand the outcome of the epidemiological studies.

### Animal models to assess the impact of macrophage iron on atherosclerosis

The key role of macrophages in atherosclerosis was extensively studied in animal models. Lipid-laden foam cells are macrophages derived from circulating monocytes that migrate into the vessel wall. Inhibition of monocyte migration, by disrupting a variety of chemokine/chemokine receptor interactions, was shown to inhibit atherosclerosis development. The osteopetrotic (op) mouse, spontaneously deficient in macrophage-colony stimulating factor (M-CSF), displayed a reduction of 86% in plaque volume, demonstrating the essential role of macrophages in atherogenesis (Smith et al., 1995). Quite recently, a CD11b–diphtheria toxin receptor transgenic mouse line was generated, whereby diphtheria toxin administration conditionally ablates monocytes/macrophages (Stoneman et al., 2007). In atherogenesis experiments, diphtheria toxin markedly decreased monocyte numbers by 50% and altered plaque development and composition, reducing collagen content and necrotic core formation, thus demonstrating that monocytes/macrophages are critical for atherogenesis.

The crucial role of macrophages in atherosclerosis raised the possibility of selective intraplaque macrophage depletion achievable as a specific therapeutic intervention to counteract plaque progression. This approach now gains increasing attention in cardiovascular medicine. Several successful strategies have recently been reported to induce macrophage cell death in atherosclerotic plaques (Martinet and De Meyer, 2007). Its feasibility is currently debated and object of several studies, aimed at locally deleting macrophages, without affecting this cell type in other tissue compartments. However, local therapies can be administered only for a relatively short time, with the limitation that macrophages may reinfiltrate the plaque after treatment.

Assessment of the impact of macrophage-associated iron on atherosclerosis could eventually provide additional mechanisms/pathways that could be targeted in macrophages to prevent/reduce atherosclerosis. Animal studies were initiated to evaluate the role of iron in macrophages, thus revisiting the iron hypothesis. Although not tested in hemochromatotic mice, atherosclerosis was studied in mice with macrophage iron depletion triggered by drug administration. The pharmacological suppression of hepcidin in mice decreased macrophage iron content, and increased cholesterol efflux, thus resulting in reduced foam cell formation (Saeed et al., 2012). In particular, the reduction of macrophage-associated iron levels lowered the formation of ROS and increased the expression of cholesterol transporters, namely ABCA1 and ABCG1. This leads to improved lipid efflux by macrophages, correlating with reduced foam cell formation and atherosclerosis (Figure 2). This approach is limited by the use of a BMP signaling pathway inhibitor to achieve hepcidin suppression. BMP signaling inhibitors are in fact expected to effect on many other biological processes involved in the formation of the atherosclerotic plaque, other than those directly dependent on hepcidin reduction. Future studies that apply drugs that directly and specifically reduce hepcidin expression or that counteract its activity are needed to examine whether hepcidin suppression by itself affects progression of atherosclerosis.

In agreement with these findings, hepcidin recently emerged as a positive regulator of atherosclerotic plaque destabilization, via regulating macrophage iron homeostasis (Li et al., 2012). Hepcidin production in the carotid artery was achieved by adenoviral infection in a mouse model of accelerated atherosclerosis. Although a change in plaque size was not observed, hepcidin overexpression significantly affected plaque composition, increasing intraplaque macrophages and decreasing VSMCs and collagen amounts. Additionally, hepcidin overexpression increased trapped iron as well as oxidized-LDL levels in intraplaque macrophages. This correlated with increased oxidative stress and expression of pro-inflammatory cytokines by macrophages and enhanced plaque vulnerability, suggesting that hepcidin plays a critical role in plaque destabilization.

Collectively, these findings indicate that the interactions of hepcidin, trapped iron, and accumulated lipids are critical for proatherosclerotic activation of macrophages leading to plaque destabilization (Figure 2). The suppression of hepcidin by specific shRNA exerts effects opposite to those reported above. These studies described a unique role for hepcidin in promoting atherosclerosis progression and plaque instability and provided evidence of a protective function of the iron-spared macrophage, at least partially clarifying the paradoxical issues observed in hemochromatosis.

A complementary approach to test the effect of iron-loaded macrophages on atherosclerosis was recently pursued (Kautz et al., 2013). Atherosclerosis was studied in the flatiron (ffe) mouse (Zohn et al., 2007), a model that specifically accumulates iron in macrophages. Contrary to the refined iron hypothesis, atherosclerosis was not increased in mice with elevated macrophage iron. In addition, increased macrophage iron levels triggered by parenteral iron administration also failed to promote atherosclerosis. These findings dispute that macrophage iron loading could be an aggravating factor in the pathogenesis of atherosclerosis.

### Effects of HO-1 in animal models of atherosclerosis

The role of HO-1 in atherosclerotic lesion formation was first investigated in apoE deficient mice, overexpressing HO-1. Overexpression of HO-1 in the vasculature was achieved by direct injection of an adenovirus expressing HO-1 (Adv-HO-1) into the left ventricles of anesthetized animals. HO-1 overexpression inhibits lesion formation and reduces iron overload in apoE deficient mice (Juan et al., 2001). Reduced iron deposition in aortic tissues of Adv-HO-1-treated mice might be explained by the observation that HO-1 overexpression augments iron recycling from cells (Ferris et al., 1999). To further examine the role of HO-1 in atherogenesis, mice deficient in both HO-1 and apoE were generated. When compared to apoE deficient mice these double knock-out mice exhibited accelerated and more advanced lesion formation in response to a cholesterol rich diet (Yet et al., 2003). Interestingly, aged HO-1 knock-out mice exhibit severe aortitis and coronary arteritis with mononuclear cell infiltration accompanied by fatty streak formation, even on a standard chow diet (Ishikawa et al., 2012).

Expression of HO-1 is strongly regulated by its substrate heme, in a Bach1-mediated manner. Bach1 is a transcriptional repressor of the hmox-1 gene that becomes inactive and undergoes ubiqitination and degradation upon heme binding (Zenke-Kawasaki et al., 2007). Consequently, deletion of the bach1 gene leads to sustained HO-1 expression in various tissues. The effect of bach1 deletion in atherosclerosis was studied in Bach1 apoE double deficient mice (Watari et al., 2008). In these mice HO-1 was upregulated in the vasculature, mainly in the vascular endothelium (Watari et al., 2008). Elevated HO-1 expression was accompanied by reduced plaque area compared with that in apoE deficient mice, supporting the anti-atherogenic nature of HO-1 (Watari et al., 2008). Overexpression of HO-1 inhibited lesion progression into vulnerable plaques, whereas inhibition of HO-1 activity augmented plaque vulnerability (Cheng et al., 2009).

Biological effects of a wide variety of molecules depend on the upregulation of HO-1 by these compounds (Bach, 2005). Accordingly, there are several anti-atherosclerotic compounds that exert their protective effects via the induction of HO-1. For example the anti-oxidant probucol, has been shown to protect from atherosclerosis by a HO-1 pathway that is independent of radical scavenging in various models of vascular diseases (Wu et al., 2006). Recently, HO-1 was found to be the molecular target of Tanshinone IIA, a lipophilic bioactive compound extracted from Salvia miltiorrhiza Bunge that exert anti-atherogenic effect via suppressing cholesterol accumulation in macrophages (Liu et al., 2014). In addition, the polyphenolic compound quercetin as well attenuates endothelial dysfunction and atherosclerosis in apoE deficient mice in a HO-1 dependent manner (Shen et al., 2013).

Taken together, these results support a protective function for HO-1 in atherosclerotic lesion formation and progression.

## Effect of iron on main players in atherogenesis

### Lipid metabolism and LDL oxidation

Elevated iron stores reflected by increased plasma ferritin levels are positively correlated with the prevalence of certain diseases such as metabolic syndrome, diabetes and obesity (Jehn et al., 2004, 2007; Lecube et al., 2008; Sun et al., 2008). All of these diseases are associated with abnormal lipid metabolism, but until recently there were few studies addressing whether elevated iron levels and lipid metabolism are directly correlated. A first study showed that HH associated with primary hypertriglyceridemia (Solanas-Barca et al., 2009), which can be improved by periodic therapeutic phlebotomy (Casanova-Esteban et al., 2011). In rats with dietary iron overload a significant increase in triglycerides, free cholesterol, cholesteryl ester, and high-density lipoprotein-cholesterol levels was observed (Brunet et al., 1999). By contrast, intraperitoneal injection of iron-dextrane enhanced serum triglyceride levels but not serum cholesterol levels in an independent study (Silva et al., 2008). Excess iron directly modulates activities of several key enzymes for cholesterol and triglyceride homeostasis—e.g., 3-hydroxy-3-methylglutaryl coenzyme A reductase, cholesterol 7alpha-hydroxylase, acyl-CoA: cholesterol acyltransferase and lipoprotein lipase - which might explain perturbations of lipid metabolism in conditions of iron overload (Brunet et al., 1999).

Other than affecting lipid metabolism, iron mediates the oxidative modification of LDL, a clear contributing factor to the pathogenesis of atherosclerosis (Heinecke et al., 1984). The molecular mechanism of iron-catalyzed LDL oxidation was extensively studied. Redox active iron that undergoes oxidation and reduction is an absolute necessity to catalyze lipid peroxidation (Lynch and Frei, 1993; Miller et al., 1993). Iron-mediated oxidation of LDL is dependent on superoxide anion (O^−•^_2_) that acts as a Fe^3+^ reducing agent, but requires neither H_2_O_2_ nor production of hydroxyl radical (OH^•^) by the Fenton reaction (Lynch and Frei, 1993).

The unlikely existence of iron in free catalytically active form in normal body fluids initiated the search for physiologically more relevant iron compounds with the ability to oxidize LDL. In fact most of the iron in the human body is found in heme that serves as a prosthetic group in Hb and other heme proteins. This ubiquitous iron compound is a very efficient catalyst of LDL oxidation (Balla et al., 1991a). Studies revealed that initiation and propagation of heme-induced lipid-peroxidation is independent of Fenton chemistry similarly to that of iron-mediated LDL oxidation. The initial interaction between heme and H_2_O_2_ might lead to the formation of ferryl and perferryl radicals, those can be responsible for initiating lipid peroxidation (Klouche et al., 2004). During heme-mediated LDL oxidation, oxidative scission of the heme ring occurs and iron is released (Balla et al., 1991a). Both heme degradation and LDL oxidation are effectively inhibited by lipid soluble antioxidants and iron chelators (Balla et al., 1991a; Pocsi et al., 2008).

Several lines of evidence suggest that heme-mediated oxidation of LDL occurs *in vivo*. High amount of heme in the plasma of the HO-1 deficient boy was correlated with extensive LDL oxidation (Jeney et al., 2002). During heme-mediated LDL oxidation heme reacts with proline and arginine residues in apolipoprotein B-100 and a unique oxidation product, gamma-glutamyl semialdehyde is formed, that is subsequently reduced to 5-hydroxy-2-aminovaleric acid (HAVA). HAVA is a hallmark of heme-mediated LDL oxidation, as other agents known to trigger LDL oxidation, such as HOCl, H_2_O_2_ alone or in combination with Cu^2+^ or Fe^2+^ induce only minor HAVA formation (Julius and Pietzsch, 2005). Elevated concentrations of HAVA were found in LDL of patients with impaired glucose tolerance and with diabetes mellitus suggesting that heme-mediated LDL oxidation occurs in these patients (Julius and Pietzsch, 2005).

Cell-free Hb when oxidized releases heme and induces oxidative modification of LDL (Jeney et al., 2002). This effect was abolished by the heme-scavenging protein Hx and by Hp or cyanide, agents that either bind free heme or strengthen the heme-globin bond, highlighting the role of heme release in this process (Miller et al., 1996; Jeney et al., 2002). Recently a feed-forward process in atheromatous lesions with the interactions of atheroma lipids and cell free Hb was described. This vicious cycle includes lipid-hydroperoxide mediated oxidation of Hb, spontaneous heme release, oxidative heme scission, iron release, and further lipid peroxidation (Nagy et al., 2010; Jeney et al., 2013; Potor et al., 2013).

Collectively, these results confirm that excess iron, heme, and cell-free Hb act in an atherogenic manner.

### Endothelial cell activation and dysfunction

Upon steady-state condition, endothelial cells provide an antithrombotic and antiadhesive surface in the vasculature. Low-grade inflammation is a characteristic of the atherosclerotic lesions in which endothelial cell activation occurs, triggering vasoconstriction, thrombosis as well as leukocyte adhesion, and transmigration (Libby, 2002). This pro-inflammatory response relies on the upregulation of a variety of genes encoding vasoconstrictive, pro-thrombic, pro-inflammatory, chemotactic, and adhesive molecules (reviewed in Pober and Sessa, 2007). Redox-sensitive mechanisms involving the activation of redox-regulated transcription factor nuclear factor-kB (NF-kB) have been implicated in the expression of these vascular inflammatory molecules (Marui et al., 1993; Kunsch and Medford, 1999).

Accumulating evidences suggest the critical role of redox active iron in mediating the pro-inflammatory response in endothelial cells. Chelation of iron by DFO leads to decreased induction of E-selectin, vascular cell adhesion molecule-1 (VCAM-1), and intercellular adhesion molecule-1 (ICAM-1) in endothelial cells stimulated by tumor necrosis factor alpha (TNF alpha) (Zhang and Frei, 2003). Switching to *in vivo* models, iron chelation inhibits the lipopolysaccharide-mediated induction of soluble cellular adhesion molecules, monocyte chemoattractant protein-1 (MCP-1) and activation of NF-kB in mice (Zhang et al., 2010). In humans, iron chelation by DFO improves nitric oxide-mediated endothelium-dependent vasodilation in patients with CAD, highlighting a role for iron in impaired nitric oxide action in atherosclerosis (Duffy et al., 2001).

The direct association between excess iron and endothelial dysfunction has been established upon physiological and pathological conditions. Administration of iron into healthy individuals provoked endothelial dysfunction accompanied by increased generation of superoxide radical in whole blood (Rooyakkers et al., 2002). Hemodialysis (HD) patients who receive intravascular iron along with erythropoiesis-stimulating agents to treat functional iron deficiency and subsequent anemia, as well as iron-overload patients, provide a unique opportunity to study the effect of iron on vascular function. There are conflicting data regarding the effect of iron on vascular function, cardiovascular risk and overall mortality in HD patients. Serum ferritin was reported as a marker of mortality in HD patients, but whether ferritin levels were regulated by iron in these patients is not clear (Kalantar-Zadeh et al., 2001). High serum ferritin level in HD patients (>600 μg/L) is associated with increased overall 4-year mortality even in the absence of infection (Kletzmayr and Horl, 2002). A cohort study concluded that iron supplementation at a dose of 1000 mg or less over 6 month does not have any adverse effect, whereas iron supplementation at higher doses is associated with elevated morbidity (Feldman et al., 2004).

Recently, more mechanistic studies were performed to show the involvement of endothelial dysfunction in iron-triggered cardiovascular complications. Intravenous administration of iron increased the levels of circulating soluble adhesion molecules in HD patients which was associated with higher risks for cardiovascular events (Kuo et al., 2012). Consistently, endothelial cells treated with iron sucrose, a widely used iron drug, changed their morphology and showed an increased ability to recruit monocyte (Kamanna et al., 2012). Iron sucrose treatment causes marked reduction in acetylcholine-mediated relaxation in rat aorta rings, thus further confirming the detrimental effect of iron on endothelial function (Kamanna et al., 2012). Iron overload diseases are associated with the presence of NTBI in the serum. In serum from hemochromatosis patients, NTBI levels were found to be positively correlated with the expressions of adhesion molecules, ICAM-1, VCAM-1, and E-selectin but not to the inflammatory marker CRP (Kartikasari et al., 2006).

Hemolytic diseases are also associated with endothelial dysfunction, therefore several studies addressed whether cell free Hb or heme can harm endothelial cells directly in these pathologies. Heme strongly sensitizes endothelial cells to oxidant-mediated killing and its plasma scavenger, Hx, completely inhibits this effect (Balla et al., 1991b). Hb when oxidized to metHb can transfer heme to the endothelium and exert the same sensitizing effect as free heme (Balla et al., 1993). More recently globin-globin cross-linked Hb multimers were identified in complicated atherosclerotic lesions (Nagy et al., 2010). The formation of these species can be triggered by inorganic and organic peroxides and involves the generation of ferrylHb and globin radicals (reviewed in Jeney et al., 2013). Interestingly, these globin-globin cross-linked Hb multimers are the exclusive species inducing pro-inflammatory response in endothelial cells *in vitro*. As a pro-inflammatory agonist, globin-globin cross-linked Hb multimers trigger the formation of intercellular gaps disrupting the integrity of the endothelial cell monolayer, induce the expression of adhesion molecules, E-selectin, ICAM-1, and VCAM-1 leading to increased monocyte adhesion (Silva et al., 2009; Potor et al., 2013).

Recently, the study of mouse models of hemolytic diseases (β-thalassemia and sickle cell disease mice) proved that heme largely contributes to endothelial activation and dysfunction and cardiovascular alterations (Tolosano et al., 2010; Vinchi and Tolosano, 2013). These effects can be strongly counteracted by the administration of an Hx-based therapy (Vinchi et al., 2008, 2013). Most of these effects have been described to rely on heme ability to activate TLR4 in endothelial cells. Heme-mediated TLR4 activation leads to Weibel-Palade body (WPB) mobilization and degranulation, thus promoting the expression of P-selectin and VWF, and NF-κB activation in endothelial cells *in vitro* and vessel wall surfaces *in vivo* (Belcher et al., 2014). By activating TLR4 pathway, heme triggers vascular stasis and occlusion, common complications associated with hemolytic disorders such as sickle cell disease. TLR4-null mice transplanted with sickle bone marrow do not exhibit heme-induced vaso-occlusion and activation of WPB/NF-κB. The ability of Hb and heme to induce stasis is abolished by the administration of the Hb and heme scavengers, Hp and Hx in a mouse model of SCD (Belcher et al., 2014). In addition heme has been recently described as a trigger of the acute chest syndrome, one of the major complications associated with SCD. In a sickle mouse model, respiratory failure due to ACS was avoided by treatment with recombinant Hx. The activation of TLR4 by heme in vascular tissues was likely responsible for this lethal type of acute lung injury. Pharmacologic inhibition of TLR4 protected sickle mice from heme-induced ACS (Ghosh et al., 2013).

These recent findings highlight a crucial role for the TLR4-activated signaling pathway in Hb/heme-mediated activation of endothelial cells and macrophages. From the point of view of atherosclerosis, a role for TLR4 in the initiation and progression of the disease is widely recognized. TLR4 is expressed on the cell surface of the main cell types involved in atherosclerosis, endothelial cells, platelets and macrophages. Its activation is required to enhance the expression of adhesion molecules and cytokines (e.g., MCP1), thus promoting the recruitment of monocytes and initiating the inflammatory response. The enhanced cytokine and chemokine release by TLR4 activation could stimulate EC and VSMC migration and proliferation, thus accelerating plaque progression (Pasterkamp et al., 2004). Additionally, oxLDL up-regulate TLR4 expression and induce cytokine expression at least partially via TLR4 activation (Pasterkamp et al., 2004; den Dekker et al., 2010). Also platelets participate in atherogenesis and show clear signs of increased activity in individuals with established cardiovascular and thrombotic disease. Increased activation of platelets via TLR4 binding could increase the risk of atherosclerosis and thrombosis (Jayachandran et al., 2010) and heme could potentially promote this event. Some mouse models and human studies also support a role of TLR4 in the progression of atherosclerotic disease. Individuals with TLR4 deficiency may be at increased risk for infection but at lower risk for cardiovascular disease (Jayachandran et al., 2010). Besides heme scavenging by Hp and Hx, targeting TLR4 as a signaling receptor downstream of heme could be an alternative therapeutic approach to reduce heme-driven pro-atherogenic effects.

Heme and oxidized Hb species can also threaten vascular endothelial cell integrity indirectly by their ability to mediate the oxidative modification of LDL (reviewed in Balla et al., 2005). Lipid hydroperoxides are transiently formed during LDL oxidation and responsible mostly for oxLDL-mediated endothelial damage and for initiation of redox signaling (Nagy et al., 2005, reviewed in Chapple et al., 2013).

Altogether these finding indicate that excess iron, extracellular Hb and heme have detrimental effects on the vascular endothelium leading to endothelial dysfunction.

### The effect of iron on macrophage polarization and function

During atherogenesis, blood monocytes are recruited to the vascular endothelium and attracted to the subendothelial space where the deposition of LDL occurs. These monocytes are later differentiated into macrophages and foam cells. Atherosclerosis macrophages are one of the most important cell populations, as they contribute to the progression of the lesions.

Macrophages are innate immune system cells therefore they exhibit great plasticity. Different stimuli and environments can lead to diverse phenotypes. Their functions comprise inflammatory responses, antimicrobial activity, tissue remodeling and iron recycling (Khallou-Laschet et al., 2010; Leitinger and Schulman, 2013).

Macrophages are key players in the regulation of iron homeostasis as they recycle 20–25 mg of iron per day from senescent erythrocytes. Macrophages engulf aged or damaged erythrocytes and catabolize heme via HO-1 activity. Heme-derived iron is then exported from phagocytic vesicles by the natural resistance-associated macrophage protein 1 (NRAMP1) and divalent metal transporter 1 (DMT1) expressed within phagolysosomal membranes. Iron is either stored coupled to ferritin or exported as ferrous iron via FPN, the only known iron exporter (Hentze et al., 2010). Interestingly, several studies demonstrated that much of the iron within plaques is associated with macrophages and foam cells. The exact source of iron still needs to be elucidated. However, it is well known that an important contribution is made by Hb-contained iron that is released from microhemorrhage within the plaque (Boyle et al., 2009; Saeed et al., 2012).

**Figure 3:**
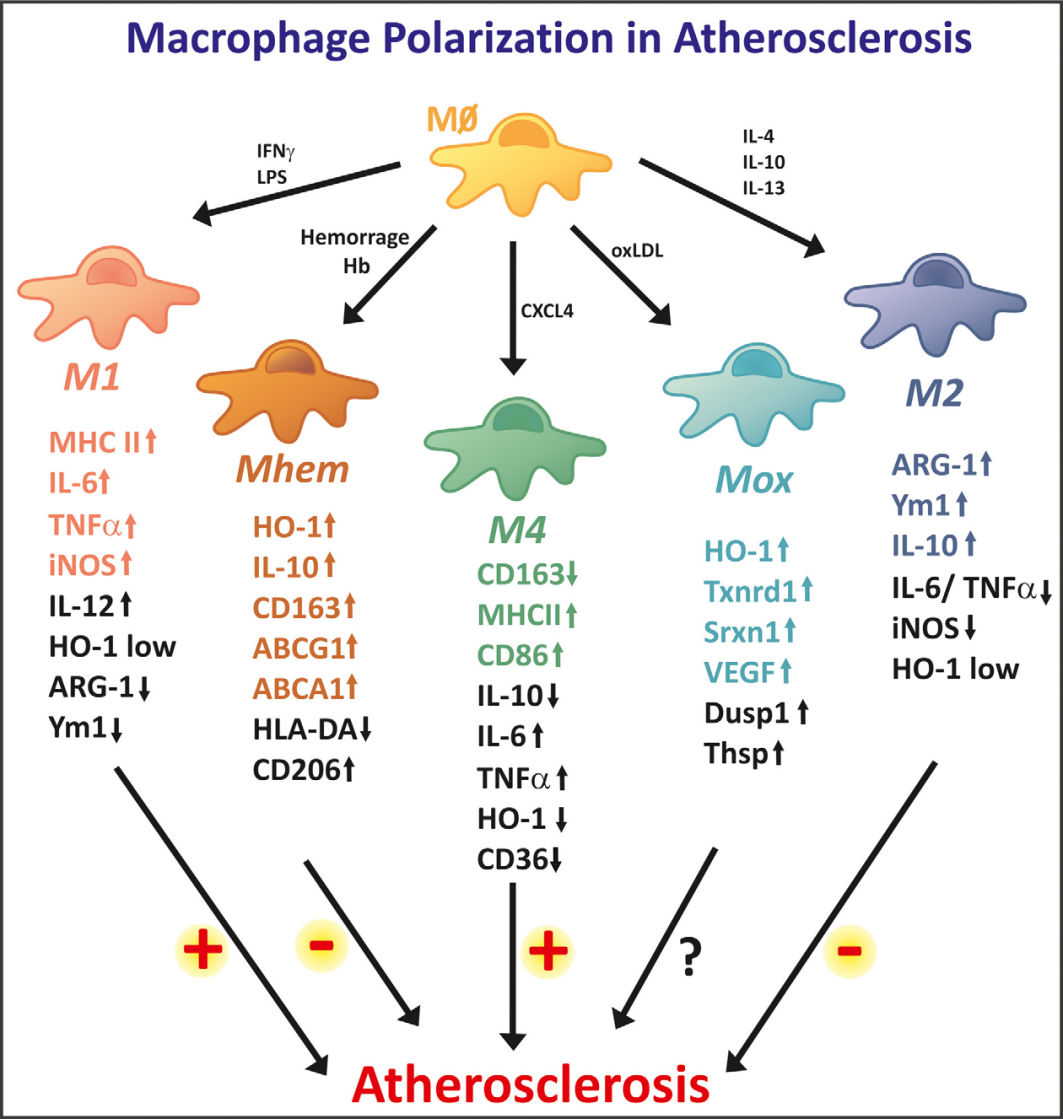
**High macrophage plasticity in atherosclerosis**. In the atherosclerotic plaque, macrophages differentiate into different phenotypes. The two extreme phenotypes are represented by M1 and M2 macrophages. M1 macrophages show strong pro-inflammatory properties, thus potentially being involved in lesion progression. M1 macrophages show high expression levels of iNOS, MHCII, and inflammatory cytokines, such as IL-6 and TNF-a. M2 macrophages are considered anti-inflammatory and are involved in tissue repair and remodeling. M2 specific markers are Arginase 1, Ym1, and IL-10. The M2 phenotype is reported as anti-atherogenic. In addition, several other macrophage phenotypes are observed in the atherosclerotic plaque. Mhem macrophages originate as a consequence of intraplaque hemorrhage and are endowed with high Hb handling ability. These anti-atherogenic macrophages express high levels of the heme-degrading enzyme HO-1 and the Hp-Hb scavenger receptor CD163. Additionally, Mheme macrophages express the cholesterol exporters ABCA1 and ABCG1, thus efficiently activating reverse cholesterol efflux. Mox macrophages are generated upon oxidized phospholipid stimulation. They show anti-oxidant properties, as they express genes involved in the anti-oxidant responses such as HO-1, Txnrd1, and Srxn1. Their potentially athero-protective effect still needs to be demonstrated. M4 macrophages differentiate in response to the chemo-attractant CXCL4, thus showing pro-inflammatory and pro-atherogenic effects. These macrophages express low levels of CD163 and high levels of MHCII and CD86. M1 and M4 macrophages promote, while M2 and Mhem macrophages counteract foam cell formation, thus having opposite effect on atherosclerosis progression.

The identification of different macrophage subtypes that polarize in response to a specific microenvironment (Leitinger and Schulman, 2013), in both human and murine atherosclerotic lesions, raised the possibility that iron itself could affect macrophage plasticity. A putative involvement of iron in the polarization of some macrophage subtypes has been recently demonstrated in atherosclerosis (Figure 3).

Two major subtypes of macrophages have been extensively studied and described: the classical activation (M1) and the alternative activation (M2) macrophages (Figure 3). M1 macrophages are polarized after exposure to IFNγ and/or microbial products such as LPS. These macrophages are characterized by a strong pro-inflammatory activity with the production of several inflammatory cytokines: IL-1β, IL-6, IL-8, IL-12, and TNFα (Butcher and Galkina, 2012). In terms of iron metabolism, M1 macrophages are prone to a low turn-over of iron with low expression of CD163, HO-1, FPN and high expression of ferritin, suggesting an iron retention phenotype with decreased iron recycling and export capacity (Recalcati et al., 2010). In chronic venous leg ulcers and wound healing models, macrophage iron overload induces an unrestrained pro-inflammatory M1 phenotype, via enhanced production of TNFα and hydroxyl radicals, suggesting that iron accumulation in macrophages contributes to a pro-inflammatory phenotype (Sindrilaru et al., 2011). Similarly, macrophage exposure to heme could lead to a pro-inflammatory activation of these cells. In fact, heme has been described as an extracellular signaling molecule able to affect the innate immune response thanks to its ability to bind and activate TLR4. By activating TLR4 heme, induces the secretion of (TNF-alpha) by macrophages (Figueiredo et al., 2007), suggesting that heme retains the ability to polarize macrophages toward an M1 rather an M2 phenotype. Whether plaque-associated macrophages are polarized toward the M1 or M2 phenotype in hemolytic sickle animal models or patients still needs to be investigated to address this point.

In atherosclerosis M1 macrophages were detected in both human and mouse lesions, in the lipid core of the plaque. M1 macrophages were the prevalent macrophage subtype in advanced lesions (Khallou-Laschet et al., 2010). It is postulated that M1 macrophages might contribute to the formation of the necrotic core, since inflammatory macrophages are prone to evolve in foam cells, eventually leading to apoptosis and cell content release. Moreover, the release of TNFα, IL-1β, IL-6 and other inflammatory cytokines by M1 macrophages in the lesion environment may contribute to the activation of endothelial cells (increasing the expression of LFA-1, VCAM-1, ICAM-1, CCL2, CD62P, and CD62E) and smooth muscle cells (increasing the expression of CCL2, CCL9, CX3CL1, CXCL10, CXCL16, and VCAM-1) and an overall increase in oxidative stress by the production of reactive oxygen and nitrogen species. In addition, M1 macrophages are associated with the response of Th1 lymphocytes, which is in accordance to an increased inflammatory response (Butcher and Galkina, 2012). All these events are expected to promote atherosclerotic plaque progression.

The alternative M2 macrophages are polarized after exposure to IL-4 or IL-13 and display an anti-inflammatory phenotype. M2-like macrophages have been described in wound healing as well as in association with tumors and with human carotid atherosclerotic plaques (Bouhlel et al., 2007). This subtype of macrophages has enhanced capacity for phagocytosis, tissue remodeling and matrix metalloproteases production (Martinez et al., 2006; Mosser and Edwards, 2008). In contrast to M1, M2 macrophages have higher expression of CD163, HO-1 and FPN and low expression of ferritin, suggesting that these macrophages have an iron release phenotype with increased iron uptake, recycling and export but low iron retention (Recalcati et al., 2010; Cairo et al., 2011). In atherosclerosis, M2 macrophages are mainly found in early lesions and are characterized by the expression of CD68 and mannose receptor (Chinetti-Gbaguidi et al., 2011). They preferentially localize in the area of the plaque overlying the lipid core (Khallou-Laschet et al., 2010). M2 macrophages are less susceptible to become foam cells and they also display a lower ability to handle lipids and to export cholesterol, due to the downregulation of the cholesterol exporter ABCA1 and the LDL carrier apoE. Also upregulation of genes involved in phagocytosis suggests that M2 macrophages in atherosclerosis have an enhanced phagocytic activity by clearing up cellular debris and dead cells (Chinetti-Gbaguidi et al., 2011). M2 macrophages are associated with a Th2 type response (Butcher and Galkina, 2012). These macrophages do not contribute to the activation of endothelial cells or smooth muscle cells since they have anti-inflammatory properties (Kleemann et al., 2008). Altogether—less susceptibility to become foam cells, high phagocytic activity and anti-inflammatory properties—place these macrophages as protective for the atherosclerotic lesion development.

Recently, new subtypes of macrophages have been described in the context of atherosclerosis, supporting the idea of an increasing diversity of macrophage subsets within the lesions.

The platelet-derived chemokine CXCL4 promotes the differentiation of monocytes to macrophages toward an M4 macrophage subtype (Gleissner et al., 2010b) (Figure 3). There is no doubt that CXCL4 is important for atherosclerosis since the deletion of the PF4 gene that encodes CXCL4 reduces atherosclerotic lesions in apoE deficient mice (Sachais et al., 2007). M4 macrophages display a distinct transcriptome when compared to M1 and M2 macrophages. The major characteristic of this subtype relies on the downregulation of CD163, the Hp-Hb scavenger receptor, which indicates that M4 macrophages are not able to clear Hb after plaque hemorrhage (Gleissner et al., 2010a,b). The incapacity of Hb uptake is consistent with the absence of HO-1 upregulation which has a protective and anti-inflammatory effect in atherosclerotic lesion (Gleissner, 2012). This also might have some implication for iron handling but further studies are necessary to characterize this macrophage subtype regarding iron turnover. In addition M4 macrophages showed reduced expression of cholesterol scavenger receptors, leading to a decreased ability to clear modified LDL. Immunohistochemistry of human post-mortem coronary arteries revealed the presence of CD68+ CD163+ as well as CD68+ CD163− macrophages, showing a correlation in the expression levels of CD163 and CXCL4 (Gleissner et al., 2010a). Whether this macrophage subtype promotes or protects against atherosclerotic plaque progression still needs to be addressed. On the basis of their reduced Hb clearance ability, a detrimental role of M4 macrophages in atherosclerosis could be speculated. Future research will be required to establish whether M4 macrophages represent a promising therapeutic target in human atherosclerosis.

Atherosclerotic lesions are characterized by the accumulation of oxidized phospholipids that also play a role in macrophage polarization. A novel macrophage phenotype denominated Mox macrophages was identified in the lesions of mice deficient for the LDL receptor (Kadl et al., 2010) (Figure 3). Mox macrophages were identified in atherosclerotic plaques in mice and accounted for 30% of all CD11b+/F4/80+ cells in established lesions. *In vitro* treatment of bone marrow-derived macrophages (BMDMs) with oxidized phospholipids reproduced differentiation toward this macrophage subtype that is distinct from both M1 and M2 subtypes. Considering the pro-oxidant action of iron, increased iron levels would be expected to enhance lipid oxidation, thus promoting Mox polarization. Whether this occurs in conditions of body iron overload has not been demonstrated.

Mox macrophages show a characteristic expression profile, including the upregulation of HO-1, thioredoxin reductase1 and sufiredoxin-1, whose expression is dependent on the redox-sensitive transcription factor Nrf2 (Kadl et al., 2010; Butcher and Galkina, 2012). These Mox-specific genes may have important functions in controlling oxidative status in an oxidizing environment and protecting cells from dying in oxidatively damaged tissue. It was demonstrated that failure of Nrf2 expression leads to various diseases related to oxidative stress, inflammation, and xenobiotic metabolism in mice. Based on these findings, a protective role of Nrf2-driven Mox macrophages in atherogenesis would be expected. Surprisingly, a recent study showed that Nrf2-null mice were protected against diet-induced atherosclerosis. Whether these Nrf2-driven Mox macrophages contribute to the initiation or progression of atherosclerotic lesion formation remains to be investigated.

Intraplaque hemorrhage is one of the key events in advanced atherosclerotic lesions leading to iron accumulation and increased oxidative stress, thus contributing to lesion development. Erythrophagocytosis is an important source of iron in plaque-associated macrophages and increased ferritin correlates with macrophage infiltration in human atheroma (Yuan et al., 1996). The recent description of hemorrhage-associated macrophages in atherosclerotic lesions further confirmed that hemorrhage-derived Hb is a source of iron for intraplaque macrophages and directs their polarization into a specialized phenotype, able to handle high Hb/iron amount (Boyle et al., 2009; Finn et al., 2012). These macrophages show high Hb handling capacity and anti-atherogenic properties and were named Hemorrhage-associated macrophages (HA-mac), Hb-stimulated macrophages, M(Hb) or heme-directed macrophages (M-hem) (Figure 3).

Macrophages associated with hemorrhage areas were characterized as CD163 high and HLA-DRlow (Boyle et al., 2011b). Moreover, as a consequence of enhanced Hb clearance, HA-mac macrophages have increased HO-1 and FPN expression, leading to facilitated heme catabolism and reduced intracellular free iron. Thus, they show antioxidative characteristics, increased expression of cholesterol exporters and resist foam cell formation both *in vivo* and in response to cholesterol loading. The reduction in intracellular free iron available for ROS formation causes increased expression of cholesterol exporters, via the activation of the LXRs (liver X receptors) pathways. HA-mac macrophages are distinct from the macrophages found in the lipid core and seem to play an atheroprotective role. *In vitro* stimulation of monocytes with Hb-Hp complexes showed a differentiation toward an HA-mac phenotype, suggesting that Hb released upon hemorrhage might model monocytes recruited to the lesion toward a specific HA-mac subtype (Boyle et al., 2011b). After treatment of human blood monocytes with heme HO-1and CD163 are upregulated, a process depending onNrf2 and the activating transcription factor 1 (Boyle et al., 2011a). Altogether, these findings suggest that iron-spared macrophages may have a protective role, as postulated by Sullivan, and that the pharmacological manipulation of iron homeostasis may be a promising target to increase macrophage reverse cholesterol transport, thus limiting atherosclerosis.

Mhem macrophages exemplify how iron can affect macrophage differentiation and function, in such a way that they can handle large amounts of Hb and iron, thus limiting iron-mediated oxidative effects and preventing lesion progression.

In atherosclerosis, macrophage activity and iron metabolism might be intrinsically connected. It is interesting to note that macrophage polarization is driven according to the specific microenvironment of the atherosclerotic lesion. The description of the different macrophage subtypes reported above suggests that also iron, in the form of Hb or via LDL oxidation, can differentially affect macrophage polarization. How broad is the range of macrophage subtypes generated in response to iron and how these subtypes contribute to atherosclerosis progression is not clear yet. Further studies are required to estimate the contribution of different iron sources to macrophage polarization and their impact on the atherosclerosis process.

### The effect of iron on VSMC phenotype switch

VSMC are the predominant cell type of the medial layer of the vessel wall. Under physiological conditions, VSMC show high contractility and a low proliferation rate. These properties are essential for VSMC to perform its primary function, contraction and dilatation of vessels to regulate blood pressure and flow. However, VSMC are not terminally differentiated cells but show the capacity to switch to synthetic, inflammatory, osteochondrogenic or macrophage-like, phenotypes upon certain stimuli. The synthetic phenotype is characterized by loss of contractility, increased motility and high proliferation rate (Campbell and Campbell, 1985). Synthetic VSMC are involved in fibrous cap formation during atherogenesis. Inflammatory VSMC phenotype is defined by cytokine secretion (e.g., IL-8, IL-6) and cell adhesion molecule expression (e.g., VCAM-1), that can regulate monocyte/macrophage adhesion and recruitment (Orr et al., 2010). Under certain pathological condition, VSMCs can undergo phenotypic transition into osteoblast-like cells, whereby they synthesize excessive extracellular matrix with parallel loss of their original function (Jono et al., 2000; Giachelli et al., 2001; Giachelli, 2003), reviewed in Sallam et al. (2013). Osteoblast specific markers are present in calcified atherosclerotic lesions, highlighting the relevance of these events in atherosclerosis (Dhore et al., 2001; Engelse et al., 2001). Finally, VSMC can differentiate into macrophage-like cells. These cells are enlarged and characterized by lipid inclusions in the cytoplasm with immunoreactivity to α-smooth muscle actin and vimentin, specific markers of VSMC. These cells are present in human atherosclerotic lesions (Vukovic et al., 2006)

Some effort was made to study the effect of iron on the phenotype switching of VSMC. Iron chelation by desferoxamine (DFO) significantly inhibited VSMC proliferation, a hallmark of the synthetic phenotype *in vitro* (Porreca et al., 1994; Wong et al., 2012), although opposing results show that iron decrease VSMC growth (Mueller et al., 2006). Iron chelation inhibits the pathological vascular remodeling response induced by balloon injury and pulmonary hypertension (Porreca et al., 1994; Wong et al., 2012). Accumulating evidence indicates that heme, and in particular, products of heme catabolism by HO-1 regulate VSMC growth (reviewed in Durante, 2003). Carbon monoxide directly inhibits VSMC proliferation by arresting cells in the G_0_/G_1_ phase of the cell cycle, whereas biliverdin and bilirubin induce VSMC apoptosis (Morita et al., 1997; Liu et al., 2002; Peyton et al., 2002).

Recently, by studying the effect of iron on osteochondrogenic differentiation of VSMC, iron was reported to inhibit inorganic phosphate (Pi)-mediated osteoblastic transition and subsequent mineralization of VSMCs *in vitro* (Zarjou et al., 2009). Importantly, iron inhibited the Pi-mediated increase in the expression of core binding factor-1 (Cbfa-1), the key osteoblast-specific transcription factor orchestrating the production of osteoblast-specific proteins, such as alkaline phosphatase and osteocalcin (Zarjou et al., 2009). Ferritin was identified as the major protective molecule behind iron-mediated inhibition of mineralization. The inhibitory effect of ferritin is strictly dependent on its ferroxidase activity but not on its iron-storage ability (Zarjou et al., 2009). Although a direct evidence of a role for iron in calcification *in vivo* is lacking, recently it has been described that iron and calcium show a highly significant spatial inverse correlation within the atherosclerotic lesions (Rajendran et al., 2012).

Although increasing evidence suggests the critical role of VSMC phenotype switch in atherogenesis, the role of iron in these mechanisms still remains to be elucidated. Further *in vitro* and *in vivo* studies are essential to clarify the particular role of iron in differentiation of VSMC into synthetic, inflammatory, osteochondrogenic, or macrophage-like phenotypes.

## Conclusive remarks

Over the last 30 years, several studies in animals and humans assessed the effect of increased body iron levels on atherosclerosis, yielding conflicting results. In the last decade, our understanding of Hb and iron biology underwent a radical revision. This significantly helped in understanding the atherogenic effects of iron and iron-containing molecules. Numerous experiments support the idea that oxidized Hb, Heme, and iron—by interacting with plaque lipids, promoting endothelial dysfunction, dictating macrophage polarization, modulating VSMC phenotype and proliferation—may affect the atherogenic process. Complex systems have evolved to control and dispose cell-free Hb, heme, and iron but these systems may be eventually overwhelmed upon excessive hemorrhage or hemolysis and upon pathological iron overload. However, to date, the impact of iron on atherosclerosis is still debated. Future studies are required to clearly address whether iron overload is a risk factor for atherosclerosis and what iron source - systemic, tissue or macrophage iron—mainly affects the atherosclerotic process. Comprehensive understanding the role of iron on atherogenesis may lead to the development of improved diagnostics and therapeutics meant to interrupt the pathologic actions of excess iron.

## Author contributions

All authors contributed to the conception and design of this review. Viktória Jeney, Francesca Vinchi, and Milene Costa Da Silva wrote the manuscript, designed and made the figures. The manuscript was critically revised by Martina U. Muckenthaler, József Balla, and György Balla.

### Conflict of interest statement

The authors declare that the research was conducted in the absence of any commercial or financial relationships that could be construed as a potential conflict of interest.

## References

[B1] Aessopos A. Farmakis D. Tsironi M. Diamanti-Kandarakis E. Matzourani M. Fragodimiri C. (2007). Endothelial function and arterial stiffness in sickle-thalassemia patients. Atherosclerosis 191, 427–432 10.1016/j.atherosclerosis.2006.04.015 16712855

[B2] Ahluwalia N. Genoux A. Ferrieres J. Perret B. Carayol M. Drouet L. (2010). Iron status is associated with carotid atherosclerotic plaques in middle-aged adults. J. Nutr. 140, 812–816 10.3945/jn.109.110353 20181783 PMC3140217

[B3] Alayash A. I. (2011). Haptoglobin: old protein with new functions. Clin. Chim. Acta 412, 493–498 10.1016/j.cca.2010.12.011 21159311

[B4] Araujo J. A. Romano E. L. Brito B. E. Parthe V. Romano M. Bracho M. (1995). Iron overload augments the development of atherosclerotic lesions in rabbits. Arterioscler. Thromb. Vasc. Biol. 15, 1172–1180 10.1161/01.ATV.15.8.1172 7542998

[B5] Bach F. H. (2005). Heme oxygenase-1: a therapeutic amplification funnel. FASEB J. 19, 1216–1219 10.1096/fj.04-3485cmt 16051687

[B9a] Bagheri B. Shokrzadeh M. Mokhberi V. Azizi A. Khalilian A. Akbari N. (2013). Association between serum iron and the severity of coronary artery disease. I. Cardiovasc. Res. J. 7, 95–98 24757630 PMC3987436

[B6] Balla G. Jacob H. S. Eaton J. W. Belcher J. D. Vercellotti G. M. (1991a). Hemin: a possible physiological mediator of low density lipoprotein oxidation and endothelial injury. Arterioscler. Thromb. 11, 1700–1711 10.1161/01.ATV.11.6.1700 1931871

[B7] Balla G. Vercellotti G. M. Muller-Eberhard U. Eaton J. Jacob H. S. (1991b). Exposure of endothelial cells to free heme potentiates damage mediated by granulocytes and toxic oxygen species. Lab. Invest. 64, 648–655 2030579

[B8] Balla J. Jacob H. S. Balla G. Nath K. Eaton J. W. Vercellotti G. M. (1993). Endothelial-cell heme uptake from heme proteins: induction of sensitization and desensitization to oxidant damage. Proc. Natl. Acad. Sci. U.S.A. 90, 9285–9289 10.1073/pnas.90.20.9285 8415693 PMC47552

[B9] Balla J. Vercellotti G. M. Jeney V. Yachie A. Varga Z. Eaton J. W. (2005). Heme, heme oxygenase and ferritin in vascular endothelial cell injury. Mol. Nutr. Food Res. 49, 1030–1043 10.1002/mnfr.200500076 16208635

[B10] Belcher J. D. Chen C. Nguyen J. Milbauer L. Abdulla F. Alayash A. I. (2014). Heme triggers TLR4 signaling leading to endothelial cell activation and vaso-occlusion in murine sickle cell disease. Blood 123, 377–390 10.1182/blood-2013-04-495887 24277079 PMC3894494

[B11] Belcher J. D. Marker P. H. Geiger P. Girotti A. W. Steinberg M. H. Hebbel R. P. (1999). Low-density lipoprotein susceptibility to oxidation and cytotoxicity to endothelium in sickle cell anemia. J. Lab. Clin. Med. 133, 605–612 10.1016/S0022-2143(99)90191-9 10360636

[B12] Borgna-Pignatti C. Rugolotto S. De Stefano P. Zhao H. Cappellini M. D. Del Vecchio G. C. (2004). Survival and complications in patients with thalassemia major treated with transfusion and deferoxamine. Haematologica 89, 1187–1193 15477202

[B13] Bouhlel M. A. Derudas B. Rigamonti E. Dievart R. Brozek J. Haulon S. (2007). PPARgamma activation primes human monocytes into alternative M2 macrophages with anti-inflammatory properties. Cell Metab. 6, 137–143 10.1016/j.cmet.2007.06.010 17681149

[B14] Boyle J. J. Harrington H. A. Piper E. Elderfield K. Stark J. Landis R. C. (2009). Coronary intraplaque hemorrhage evokes a novel atheroprotective macrophage phenotype. Am. J. Pathol. 174, 1097–1108 10.2353/ajpath.2009.080431 19234137 PMC2665768

[B15] Boyle J. J. Johns M. Kampfer T. Nguyen A. T. Game L. Schaer D. J. (2011a). Activating transcription factor 1 directs Mhem atheroprotective macrophages through coordinated iron handling and foam cell protection. Circ. Res. 110, 20–33 10.1161/CIRCRESAHA.111.247577 22052915

[B16] Boyle J. J. Johns M. Lo J. Chiodini A. Ambrose N. Evans P. C. (2011b). Heme induces heme oxygenase 1 via Nrf2: role in the homeostatic macrophage response to intraplaque hemorrhage. Arterioscler. Thromb. Vasc. Biol. 31, 2685–2691 10.1161/ATVBAHA.111.225813 21868703

[B17] Brissot P. Ropert M. Le Lan C. Loreal O. (2012). Non-transferrin bound iron: a key role in iron overload and iron toxicity. Biochim. Biophys. Acta 1820, 403–410 10.1016/j.bbagen.2011.07.014 21855608

[B18] Brizzi P. Isaja T. D'agata A. Malaguarnera L. Malaguarnera M. Musumeci S. (2002). Oxidized LDL antibodies (OLAB) in patients with beta-thalassemia major. J. Atheroscler. Thromb. 9, 139–144 10.5551/jat.9.139 12226555

[B18a] Brouwers A. Langlois M. Delanghe J. Billiet J. De Buyzere M. Vercaemst R. (2004). Oxidized low-density lipoprotein, iron stores, and haptoglobin polymorphism. Atherosclerosis 176, 189–195 10.1016/j.atherosclerosis.2004.05.005 15306193

[B19] Brunet S. Thibault L. Delvin E. Yotov W. Bendayan M. Levy E. (1999). Dietary iron overload and induced lipid peroxidation are associated with impaired plasma lipid transport and hepatic sterol metabolism in rats. Hepatology 29, 1809–1817 10.1002/hep.510290612 10347124

[B20] Butcher M. J. Galkina E. V. (2012). Phenotypic and functional heterogeneity of macrophages and dendritic cell subsets in the healthy and atherosclerosis-prone aorta. Front. Physiol. 3:44 10.3389/fphys.2012.00044 22457649 PMC3307136

[B21] Cairo G. Recalcati S. Mantovani A. Locati M. (2011). Iron trafficking and metabolism in macrophages: contribution to the polarized phenotype. Trends Immunol. 32, 241–247 10.1016/j.it.2011.03.007 21514223

[B22] Campbell G. R. Campbell J. H. (1985). Smooth muscle phenotypic changes in arterial wall homeostasis: implications for the pathogenesis of atherosclerosis. Exp. Mol. Pathol. 42, 139–162 10.1016/0014-4800(85)90023-1 3884359

[B23] Carlier S. Kakadiaris I. A. Dib N. Vavuranakis M. O'malley S. M. Gul K. (2005). Vasa vasorum imaging: a new window to the clinical detection of vulnerable atherosclerotic plaques. Curr. Atheroscler. Rep. 7, 164–169 10.1007/s11883-005-0040-2 15727733

[B24] Casanova-Esteban P. Guiral N. Andres E. Gonzalvo C. Mateo-Gallego R. Giraldo P. (2011). Effect of phlebotomy on lipid metabolism in subjects with hereditary hemochromatosis. Metab. Clin. Exp. 60, 830–834 10.1016/j.metabol.2010.07.035 20846699

[B25] Chan K. H. Ng M. K. Stocker R. (2011). Haem oxygenase-1 and cardiovascular disease: mechanisms and therapeutic potential. Clin. Sci. 120, 493–504 10.1042/CS20100508 21355854

[B26] Chapple S. J. Cheng X. Mann G. E. (2013). Effects of 4-hydroxynonenal on vascular endothelial and smooth muscle cell redox signaling and function in health and disease. Redox Biol. 1, 319–331 10.1016/j.redox.2013.04.001 24024167 PMC3757694

[B27] Cheng C. Noordeloos A. M. Jeney V. Soares M. P. Moll F. Pasterkamp G. (2009). Heme oxygenase 1 determines atherosclerotic lesion progression into a vulnerable plaque. Circulation 119, 3017–3027 10.1161/CIRCULATIONAHA.108.808618 19487598

[B28] Cheung Y. F. Chan G. C. Ha S. Y. (2002). Arterial stiffness and endothelial function in patients with beta-thalassemia major. Circulation 106, 2561–2566 10.1161/01.CIR.0000037225.92759.A7 12427652

[B29] Cheung Y. F. Chan G. C. Ha S. Y. (2008). Effect of deferasirox (ICL670) on arterial function in patients with beta-thalassaemia major. Br. J. Haematol. 141, 728–733 10.1111/j.1365-2141.2008.07092.x 18318756

[B30] Cheung Y. F. Chow P. C. Chan G. C. Ha S. Y. (2006). Carotid intima-media thickness is increased and related to arterial stiffening in patients with beta-thalassaemia major. Br. J. Haematol. 135, 732–734 10.1111/j.1365-2141.2006.06349.x 17107355

[B31] Chinetti-Gbaguidi G. Baron M. Bouhlel M. A. Vanhoutte J. Copin C. Sebti Y. (2011). Human atherosclerotic plaque alternative macrophages display low cholesterol handling but high phagocytosis because of distinct activities of the PPARgamma and LXRalpha pathways. Circ. Res. 108, 985–995 10.1161/CIRCRESAHA.110.233775 21350215 PMC3319502

[B32] Costacou T. Levy A. P. (2012). Haptoglobin genotype and its role in diabetic cardiovascular disease. J. Cardiovasc. Transl. Res. 5, 423–435 10.1007/s12265-012-9361-z 22447230 PMC3595557

[B33] Dabbagh A. J. Shwaery G. T. Keaney J. F. Jr. Frei B. (1997). Effect of iron overload and iron deficiency on atherosclerosis in the hypercholesterolemic rabbit. Arterioscler. Thromb. Vasc. Biol. 17, 2638–2645 10.1161/01.ATV.17.11.2638 9409237

[B34] de Chadarevian J. P. Balarezo F. S. Heggere M. Dampier C. (2001). Splenic arteries and veins in pediatric sickle cell disease. Pediatr. Dev. Pathol. 4, 538–544 10.1007/s10024001-0045-y 11826359

[B35] den Dekker W. K. Cheng C. Pasterkamp G. Duckers H. J. (2010). Toll like receptor 4 in atherosclerosis and plaque destabilization. Atherosclerosis 209, 314–320 10.1016/j.atherosclerosis.2009.09.075 19900676

[B36] Depalma R. G. Hayes V. W. Chow B. K. Shamayeva G. May P. E. Zacharski L. R. (2010). Ferritin levels, inflammatory biomarkers, and mortality in peripheral arterial disease: a substudy of the Iron (Fe) and Atherosclerosis Study (FeAST) Trial. J. Vasc. Surg. 51, 1498–1503 10.1016/j.jvs.2009.12.068 20304584

[B37] Dhore C. R. Cleutjens J. P. Lutgens E. Cleutjens K. B. Geusens P. P. Kitslaar P. J. (2001). Differential expression of bone matrix regulatory proteins in human atherosclerotic plaques. Arterioscler. Thromb. Vasc. Biol. 21, 1998–2003 10.1161/hq1201.100229 11742876

[B38] Duffy S. J. Biegelsen E. S. Holbrook M. Russell J. D. Gokce N. Keaney J. F. (2001). Iron chelation improves endothelial function in patients with coronary artery disease. Circulation 103, 2799–2804 10.1161/01.CIR.103.23.2799 11401935

[B39] Durante W. (2003). Heme oxygenase-1 in growth control and its clinical application to vascular disease. J. Cell. Physiol. 195, 373–382 10.1002/jcp.10274 12704646

[B40] Durante W. (2011). Protective role of heme oxygenase-1 against inflammation in atherosclerosis. Front. Biosci. 16, 2372–2388 10.2741/3860 21622183 PMC5940339

[B41] Eisenstein R. S. Garcia-Mayol D. Pettingell W. Munro H. N. (1991). Regulation of ferritin and heme oxygenase synthesis in rat fibroblasts by different forms of iron. Proc. Natl. Acad. Sci. U.S.A. 88, 688–692 10.1073/pnas.88.3.688 1992460 PMC50878

[B42] Elsharawy M. A. Moghazy K. M. Shawarby M. A. (2009). Atherosclerosis in sickle cell disease—a review. Int. J. Angiol. 18, 62–66 10.1055/s-0031-1278326 22477494 PMC2780857

[B43] Engelse M. A. Neele J. A. Bronckers A. L. J. J. Pannekoek H. De Vries C. J. M. (2001). Vascular calcification: expression patterns of the osteoblast-specific gene core binding factor alpha-1 and the protective factor matrix gla protein in human atherogenesis. Cardiovasc. Res. 52, 281–289 10.1016/S0008-6363(01)00375-3 11684076

[B44] Erdogan D. Gullu H. Yildirim E. Tok D. Kirbas I. Ciftci O. (2006). Low serum bilirubin levels are independently and inversely related to impaired flow-mediated vasodilation and increased carotid intima-media thickness in both men and women. Atherosclerosis 184, 431–437 10.1016/j.atherosclerosis.2005.05.011 15979081

[B45] Erkan A. Ekici B. Ugurlu M. Is G. Seker R. Demirtas S. (2013). The role of bilirubin and its protective function against coronary heart disease. Herz. [Epub ahead of print]. 10.1007/s00059-013-3872-5 23861132

[B46] Failla M. Giannattasio C. Piperno A. Vergani A. Grappiolo A. Gentile G. (2000). Radial artery wall alterations in genetic hemochromatosis before and after iron depletion therapy. Hepatology 32, 569–573 10.1053/jhep.2000.16265 10960451

[B47] Feldman H. I. Joffe M. Robinson B. Knauss J. Cizman B. Guo W. (2004). Administration of parenteral iron and mortality among hemodialysis patients. J. Am. Soc. Nephrol. 15, 1623–1632 10.1097/01.ASN.0000128009.69594.BE 15153574

[B48] Ferrara D. E. Taylor W. R. (2005). Iron chelation and vascular function: in search of the mechanisms. Arterioscler. Thromb. Vasc. Biol. 25, 2235–2237 10.1161/01.ATV.0000189303.45609.1f 16258147

[B49] Ferris C. D. Jaffrey S. R. Sawa A. Takahashi M. Brady S. D. Barrow R. K. (1999). Haem oxygenase-1 prevents cell death by regulating cellular iron. Nat. Cell Biol. 1, 152–157 10.1038/11072 10559901

[B50] Figueiredo R. T. Fernandez P. L. Mourao-Sa D. S. Porto B. N. Dutra F. F. Alves L. S. (2007). Characterization of heme as activator of Toll-like receptor 4. J. Biol. Chem. 282, 20221–20229 10.1074/jbc.M610737200 17502383

[B51] Finn A. V. Nakano M. Polavarapu R. Karmali V. Saeed O. Zhao X. (2012). Hemoglobin directs macrophage differentiation and prevents foam cell formation in human atherosclerotic plaques. J. Am. Coll. Cardiol. 59, 166–177 10.1016/j.jacc.2011.10.852 22154776 PMC3253238

[B52] Fleming R. E. Feng Q. Britton R. S. (2011). Knockout mouse models of iron homeostasis. Annu. Rev. Nutr. 31, 117–137 10.1146/annurev-nutr-072610-145117 21548776

[B53] Francis R. B. Jr. Johnson C. S. (1991). Vascular occlusion in sickle cell disease: current concepts and unanswered questions. Blood 77, 1405–1414 2009364

[B54] Gaenzer H. Marschang P. Sturm W. Neumayr G. Vogel W. Patsch J. (2002). Association between increased iron stores and impaired endothelial function in patients with hereditary hemochromatosis. J. Am. Coll. Cardiol. 40, 2189–2194 10.1016/S0735-1097(02)02611-6 12505233

[B55] Ganz T. Nemeth E. (2011). Hepcidin and disorders of iron metabolism. Annu. Rev. Med. 62, 347–360 10.1146/annurev-med-050109-142444 20887198

[B56] Ghosh S. Adisa O. A. Chappa P. Tan F. Jackson K. A. Archer D. R. (2013). Extracellular hemin crisis triggers acute chest syndrome in sickle mice. J. Clin. Invest. 123, 4809–4820 10.1172/JCI64578 24084741 PMC3809772

[B57] Giachelli C. M. (2003). Vascular calcification: *in vitro* evidence for the role of inorganic phosphate. J. Am. Soc. Nephrol. 14, S300–S304 10.1097/01.ASN.0000081663.52165.66 12939385

[B58] Giachelli C. M. Jono S. Shioi A. Nishizawa Y. Mori K. Morii H. (2001). Vascular calcification and inorganic phosphate. Am. J. Kidney Dis. 38, S34–S37 10.1053/ajkd.2001.27394 11576919

[B59] Gillum R. F. Mussolino M. E. Madans J. H. (1997). Coronary heart disease incidence and survival in African-American women and men. The NHANES I epidemiologic follow-up study. Ann. Intern. Med. 127, 111–118 10.7326/0003-4819-127-2-199707150-00003 9229999

[B60] Gleissner C. A. (2012). Macrophage phenotype modulation by CXCL4 in Atherosclerosis. Front. Physiol. 3:1. 10.3389/fphys.2012.00001 22275902 PMC3257836

[B61] Gleissner C. A. Shaked I. Erbel C. Bockler D. Katus H. A. Ley K. (2010a). CXCL4 downregulates the atheroprotective hemoglobin receptor CD163 in human macrophages. Circ. Res. 106, 203–211 10.1161/CIRCRESAHA.109.199505 19910578 PMC2876722

[B62] Gleissner C. A. Shaked I. Little K. M. Ley K. (2010b). CXC chemokine ligand 4 induces a unique transcriptome in monocyte-derived macrophages. J. Immunol. 184, 4810–4818 10.4049/jimmunol.0901368 20335529 PMC3418140

[B63] Goldenstein H. Levy N. S. Levy A. P. (2012). Haptoglobin genotype and its role in determining heme-iron mediated vascular disease. Pharmacol. Res. 66, 1–6 10.1016/j.phrs.2012.02.011 22465143 PMC3345090

[B64] Gozzelino R. Jeney V. Soares M. P. (2010). Mechanisms of cell protection by heme oxygenase-1. Annu. Rev. Pharmacol. Toxicol. 50, 323–354 10.1146/annurev.pharmtox.010909.105600 20055707

[B65] Graham J. K. Mosunjac M. Hanzlick R. L. (2007). Sickle cell lung disease and sudden death: a retrospective/prospective study of 21 autopsy cases and literature review. Am. J. Forensic Med. Pathol. 28, 168–172 10.1097/01.paf.0000257397.92466.50 17525572

[B66] Gustafsson H. Hallbeck M. Norell M. Lindgren M. Engstrom M. Rosen A. (2013). Fe(III) distribution varies substantially within and between atherosclerotic plaques. Magn. Reson. Med. 71, 885–892 10.1002/mrm.24687 23447110

[B67] Hahalis G. Kremastinos D. T. Terzis G. Kalogeropoulos A. P. Chrysanthopoulou A. Karakantza M. (2008). Global vasomotor dysfunction and accelerated vascular aging in beta-thalassemia major. Atherosclerosis 198, 448–457 10.1016/j.atherosclerosis.2007.09.030 17988670

[B68] Haidari M. Javadi E. Sanati A. Hajilooi M. Ghanbili J. (2001). Association of increased ferritin with premature coronary stenosis in men. Clin. Chem. 47, 1666–1672 11514401

[B69] Harrison P. M. Arosio P. (1996). The ferritins: molecular properties, iron storage function and cellular regulation. Biochim. Biophys. Acta 1275, 161–203 10.1016/0005-2728(96)00022-9 8695634

[B70] Heinecke J. W. Rosen H. Chait A. (1984). Iron and copper promote modification of low density lipoprotein by human arterial smooth muscle cells in culture. J. Clin. Invest. 74, 1890–1894 10.1172/JCI111609 6501577 PMC425370

[B71] Hentze M. W. Muckenthaler M. U. Galy B. Camaschella C. (2010). Two to tango: regulation of Mammalian iron metabolism. Cell 142, 24–38 10.1016/j.cell.2010.06.028 20603012

[B72] Holay M. P. Choudhary A. A. Suryawanshi S. D. (2012). Serum ferritin-a novel risk factor in acute myocardial infarction. Indian Heart J. 64, 173–177 10.1016/S0019-4832(12)60056-X 22572495 PMC3861068

[B73] Houschyar K. S. Ludtke R. Dobos G. J. Kalus U. Broecker-Preuss M. Rampp T. (2012). Effects of phlebotomy-induced reduction of body iron stores on metabolic syndrome: results from a randomized clinical trial. BMC Med. 10:54 10.1186/1741-7015-10-54 22647517 PMC3386865

[B74] Hulley S. Grady D. Bush T. Furberg C. Herrington D. Riggs B. (1998). Randomized trial of estrogen plus progestin for secondary prevention of coronary heart disease in postmenopausal women. JAMA 280, 605–613 10.1001/jama.280.7.605 9718051

[B75] Ijas P. Saksi J. Soinne L. Tuimala J. Jauhiainen M. Jula A. (2013). Haptoglobin 2 allele associates with unstable carotid plaque and major cardiovascular events. Atherosclerosis 230, 228–234 10.1016/j.atherosclerosis.2013.07.008 24075749

[B76] Ishikawa K. Navab M. Lusis A. J. (2012). Vasculitis, Atherosclerosis, and altered HDL composition in heme-oxygenase-1-knockout mice. Int. J. Hypertens. 2012:948203 10.1155/2012/948203 22518297 PMC3296294

[B77] Ishizaka N. Saito K. Mori I. Matsuzaki G. Ohno M. Nagai R. (2005). Iron chelation suppresses ferritin upregulation and attenuates vascular dysfunction in the aorta of angiotensin II-infused rats. Arterioscler. Thromb. Vasc. Biol. 25, 2282–2288 10.1161/01.ATV.0000181763.57495.2b 16100038

[B78] Jayachandran M. Miller V. M. Brunn G. J. Owen W. G. (2010). Platelet response as a sentinel marker of toll-like receptor 4 activation in mice. Thromb. Res. 126, 414–417 10.1016/j.thromres.2009.05.005 19482340 PMC2978423

[B79] Jehn M. Clark J. M. Guallar E. (2004). Serum ferritin and risk of the metabolic syndrome in U.S. adults. Diabetes Care 27, 2422–2428 10.2337/diacare.27.10.2422 15451911

[B80] Jehn M. L. Guallar E. Clark J. M. Couper D. Duncan B. B. Ballantyne C. M. (2007). A prospective study of plasma ferritin level and incident diabetes: the Atherosclerosis Risk in Communities (ARIC) Study. Am. J. Epidemiol. 165, 1047–1054 10.1093/aje/kwk093 17284722

[B81] Jeney V. Balla J. Yachie A. Varga Z. Vercellotti G. M. Eaton J. W. (2002). Pro-oxidant and cytotoxic effects of circulating heme. Blood 100, 879–887 10.1182/blood.V100.3.879 12130498

[B82] Jeney V. Eaton J. W. Balla G. Balla J. (2013). Natural history of the bruise: formation, elimination, and biological effects of oxidized hemoglobin. Oxid. Med. Cell. Longev. 2013:703571 10.1155/2013/703571 23766858 PMC3671564

[B83] Jono S. Mckee M. D. Murry C. E. Shioi A. Nishizawa Y. Mori K. (2000). Phosphate regulation of vascular smooth muscle cell calcification. Circ. Res. 87, E10–E17 10.1161/01.RES.87.7.e10 11009570

[B84] Juan S. H. Lee T. S. Tseng K. W. Liou J. Y. Shyue S. K. Wu K. K. (2001). Adenovirus-mediated heme oxygenase-1 gene transfer inhibits the development of atherosclerosis in apolipoprotein E-deficient mice. Circulation 104, 1519–1525 10.1161/hc3801.095663 11571246

[B85] Julius U. Pietzsch J. (2005). Glucose-induced enhancement of hemin-catalyzed LDL oxidation *in vitro* and *in vivo*. Antioxid. Redox Signal. 7, 1507–1512 10.1089/ars.2005.7.1507 16356114

[B86] Kadl A. Meher A. K. Sharma P. R. Lee M. Y. Doran A. C. Johnstone S. R. (2010). Identification of a novel macrophage phenotype that develops in response to atherogenic phospholipids via Nrf2. Circ. Res. 107, 737–746 10.1161/CIRCRESAHA.109.215715 20651288 PMC2941538

[B87] Kalantar-Zadeh K. Don B. R. Rodriguez R. A. Humphreys M. H. (2001). Serum ferritin is a marker of morbidity and mortality in hemodialysis patients. Am. J. Kidney Dis. 37, 564–572 10.1053/ajkd.2001.22433 11228181

[B88] Kalantar-Zadeh K. Rodriguez R. A. Humphreys M. H. (2004). Association between serum ferritin and measures of inflammation, nutrition and iron in haemodialysis patients. Nephrol. Dial. Transplant. 19, 141–149 10.1093/ndt/gfg493 14671049

[B89] Kamanna V. S. Ganji S. H. Shelkovnikov S. Norris K. Vaziri N. D. (2012). Iron sucrose promotes endothelial injury and dysfunction and monocyte adhesion/infiltration. Am. J. Nephrol. 35, 114–119 10.1159/000334939 22212390 PMC3265804

[B90] Kaneda H. Ohno M. Taguchi J. Togo M. Hashimoto H. Ogasawara K. (2002). Heme oxygenase-1 gene promoter polymorphism is associated with coronary artery disease in Japanese patients with coronary risk factors. Arterioscler. Thromb. Vasc. Biol. 22, 1680–1685 10.1161/01.ATV.0000033515.96747.6F 12377749

[B91] Kannel W. B. Hjortland M. C. Mcnamara P. M. Gordon T. (1976). Menopause and risk of cardiovascular disease: the Framingham study. Ann. Intern. Med. 85, 447–452 10.7326/0003-4819-85-4-447 970770

[B92] Kartikasari A. E. Georgiou N. A. Visseren F. L. Van Kats-Renaud H. Van Asbeck B. S. Marx J. J. (2006). Endothelial activation and induction of monocyte adhesion by nontransferrin-bound iron present in human sera. FASEB J. 20, 353–355 10.1096/fj.05-4700fje 16368718

[B93] Kautz L. Gabayan V. Wang X. Wu J. Onwuzurike J. Jung G. (2013). Testing the iron hypothesis in a mouse model of atherosclerosis. Cell Rep. 5, 1436–1442 10.1016/j.celrep.2013.11.009 24316081 PMC3880128

[B94] Khallou-Laschet J. Varthaman A. Fornasa G. Compain C. Gaston A. T. Clement M. (2010). Macrophage plasticity in experimental atherosclerosis. PLoS ONE 5:e8852 10.1371/journal.pone.0008852 20111605 PMC2810335

[B95] Kiechl S. Willeit J. (1999). The natural course of atherosclerosis. Part II: vascular remodeling. Bruneck Study Group. Arterioscler. Thromb. Vasc. Biol. 19, 1491–1498 10.1161/01.ATV.19.6.1491 10364080

[B96] Kiechl S. Willeit J. Egger G. Poewe W. Oberhollenzer F. (1997). Body iron stores and the risk of carotid atherosclerosis: prospective results from the Bruneck study. Circulation 96, 3300–3307 10.1161/01.CIR.96.10.3300 9396420

[B97] Kimm H. Yun J. E. Jo J. Jee S. H. (2009). Low serum bilirubin level as an independent predictor of stroke incidence: a prospective study in Korean men and women. Stroke 40, 3422–3427 10.1161/STROKEAHA.109.560649 19713538

[B98] Kirk E. A. Heinecke J. W. Leboeuf R. C. (2001). Iron overload diminishes atherosclerosis in apoE-deficient mice. J. Clin. Invest. 107, 1545–1553 10.1172/JCI7664 11413162 PMC200187

[B99] Kleemann R. Zadelaar S. Kooistra T. (2008). Cytokines and atherosclerosis: a comprehensive review of studies in mice. Cardiovasc. Res. 79, 360–376 10.1093/cvr/cvn120 18487233 PMC2492729

[B100] Kletzmayr J. Horl W. H. (2002). Iron overload and cardiovascular complications in dialysis patients. Nephrol. Dial. Transplant. 17 Suppl. 2, 25–29 10.1093/ndt/17.suppl_2.25 11904355

[B101] Klouche K. Morena M. Canaud B. Descomps B. Beraud J. J. Cristol J. P. (2004). Mechanism of *in vitro* heme-induced LDL oxidation: effects of antioxidants. Eur. J. Clin. Invest. 34, 619–625 10.1111/j.1365-2362.2004.01395.x 15379761

[B102] Kolodgie F. D. Gold H. K. Burke A. P. Fowler D. R. Kruth H. S. Weber D. K. (2003). Intraplaque hemorrhage and progression of coronary atheroma. N. Engl. J. Med. 349, 2316–2325 10.1056/NEJMoa035655 14668457

[B103] Kolodgie F. D. Virmani R. Burke A. P. Farb A. Weber D. K. Kutys R. (2004). Pathologic assessment of the vulnerable human coronary plaque. Heart 90, 1385–1391 10.1136/hrt.2004.041798 15547008 PMC1768577

[B104] Kremastinos D. T. Flevari P. Spyropoulou M. Vrettou H. Tsiapras D. Stavropoulos-Giokas C. G. (1999). Association of heart failure in homozygous beta-thalassemia with the major histocompatibility complex. Circulation 100, 2074–2078 10.1161/01.CIR.100.20.2074 10562263

[B105] Kristiansen M. Graversen J. H. Jacobsen C. Sonne O. Hoffman H. J. Law S. K. (2001). Identification of the haemoglobin scavenger receptor. Nature 409, 198–201 10.1038/35051594 11196644

[B106] Kunsch C. Medford R. M. (1999). Oxidative stress as a regulator of gene expression in the vasculature. Circ. Res. 85, 753–766 10.1161/01.RES.85.8.753 10521248

[B107] Kuo K. L. Hung S. C. Lin Y. P. Tang C. F. Lee T. S. Lin C. P. (2012). Intravenous ferric chloride hexahydrate supplementation induced endothelial dysfunction and increased cardiovascular risk among hemodialysis patients. PLoS ONE 7:e50295 10.1371/journal.pone.0050295 23227165 PMC3515606

[B108] Landis R. C. Philippidis P. Domin J. Boyle J. J. Haskard D. O. (2013). Haptoglobin Genotype-Dependent Anti-Inflammatory Signaling in CD163(+) Macrophages. Int. J. Inflam. 2013:980327 10.1155/2013/980327 23710416 PMC3655560

[B109] Lauffer R. B. (1990). Iron depletion and coronary disease. Am. Heart J. 119, 1448–1449 10.1016/S0002-8703(05)80216-9 2353637

[B110] Lecube A. Hernandez C. Pelegri D. Simo R. (2008). Factors accounting for high ferritin levels in obesity. Int. J. Obes. 32, 1665–1669 10.1038/ijo.2008.154 18779821

[B111] Lee H. T. Chiu L. L. Lee T. S. Tsai H. L. Chau L. Y. (2003). Dietary iron restriction increases plaque stability in apolipoprotein-e-deficient mice. J. Biomed. Sci. 10, 510–517 10.1007/BF02256112 12928591

[B112] Lee T. S. Shiao M. S. Pan C. C. Chau L. Y. (1999). Iron-deficient diet reduces atherosclerotic lesions in apoE-deficient mice. Circulation 99, 1222–1229 10.1161/01.CIR.99.9.1222 10069791

[B113] Leitinger N. Schulman I. G. (2013). Phenotypic polarization of macrophages in atherosclerosis. Arterioscler. Thromb. Vasc. Biol. 33, 1120–1126 10.1161/ATVBAHA.112.300173 23640492 PMC3745999

[B114] Li J. J. Meng X. Si H. P. Zhang C. Lv H. X. Zhao Y. X. (2012). Hepcidin destabilizes atherosclerotic plaque via overactivating macrophages after erythrophagocytosis. Arterioscler. Thromb. Vasc. Biol. 32, 1158–1166 10.1161/ATVBAHA.112.246108 22383698

[B115] Liang K. W. Sheu W. H. Lee W. L. Lee I. T. Lin S. Y. Ting C. T. (2013). Shorter GT repeats in the heme oxygenase-1 gene promoter are associated with a lower severity score in coronary artery disease. J. Chin. Med. Assoc. 76, 312–318 10.1016/j.jcma.2013.03.005 23602216

[B116] Libby P. (2002). Inflammation in atherosclerosis. Nature 420, 868–874 10.1038/nature01323 12490960

[B117] Lioupis C. Barbatis C. Drougou A. Koliaraki V. Mamalaki A. Klonaris C. (2011). Association of haptoglobin genotype and common cardiovascular risk factors with the amount of iron in atherosclerotic carotid plaques. Atherosclerosis 216, 131–138 10.1016/j.atherosclerosis.2011.01.028 21316675

[B118] Lioupis C. Barbatis C. Lazari P. Liasis N. Klonaris C. Georgopoulos S. (2012). Macrophage infiltration and smooth muscle cells content associated with haptoglobin genotype in human atherosclerotic carotid plaques. Angiology 63, 178–183 10.1177/0003319711410051 21642285

[B119] Lipiski M. Deuel J. W. Baek J. H. Engelsberger W. R. Buehler P. W. Schaer D. J. (2013). Human Hp1-1 and Hp2-2 phenotype-specific haptoglobin therapeutics are both effective *in vitro* and in guinea pigs to attenuate hemoglobin toxicity. Antioxid. Redox Signal. 19, 1619–1633 10.1089/ars.2012.5089 23418677 PMC3809386

[B120] Liu X. M. Chapman G. B. Wang H. Durante W. (2002). Adenovirus-mediated heme oxygenase-1 gene expression stimulates apoptosis in vascular smooth muscle cells. Circulation 105, 79–84 10.1161/hc0102.101369 11772880

[B121] Liu Z. Wang J. Huang E. Gao S. Li H. Lu J. (2014). Tanshinone IIA suppresses cholesterol accumulation in human macrophages: role of haem oxygenase-1. J. Lipid Res. 55, 201–213 10.1194/jlr.M040394 24302760 PMC3886659

[B122] Livrea M. A. Tesoriere L. Maggio A. D'arpa D. Pintaudi A. M. Pedone E. (1998). Oxidative modification of low-density lipoprotein and atherogenetic risk in beta-thalassemia. Blood 92, 3936–3942 9808587

[B123] Lublinghoff N. Winkler K. Winkelmann B. R. Seelhorst U. Wellnitz B. Boehm B. O. (2009). Genetic variants of the promoter of the heme oxygenase-1 gene and their influence on cardiovascular disease (the Ludwigshafen Risk and Cardiovascular Health study). BMC Med. Genet. 10:36 10.1186/1471-2350-10-36 19389234 PMC2678993

[B124] Lynch S. M. Frei B. (1993). Mechanisms of copper- and iron-dependent oxidative modification of human low density lipoprotein. J. Lipid Res. 34, 1745–1753 8245725

[B125] Manousou P. Kalambokis G. Grillo F. Watkins J. Xirouchakis E. Pleguezuelo M. (2011). Serum ferritin is a discriminant marker for both fibrosis and inflammation in histologically proven non-alcoholic fatty liver disease patients. Liver Int. 31, 730–739 10.1111/j.1478-3231.2011.02488.x 21457446

[B126] Martinet W. De Meyer G. R. (2007). Selective depletion of macrophages in atherosclerotic plaques: myth, hype, or reality? Circ. Res. 100, 751–753 10.1161/01.RES.0000263397.14481.96 17395878

[B127] Martinez F. O. Gordon S. Locati M. Mantovani A. (2006). Transcriptional profiling of the human monocyte-to-macrophage differentiation and polarization: new molecules and patterns of gene expression. J. Immunol. 177, 7303–7311 17082649 10.4049/jimmunol.177.10.7303

[B128] Marui N. Offermann M. K. Swerlick R. Kunsch C. Rosen C. A. Ahmad M. (1993). Vascular cell adhesion molecule-1 (VCAM-1) gene transcription and expression are regulated through an antioxidant-sensitive mechanism in human vascular endothelial cells. J. Clin. Invest. 92, 1866–1874 10.1172/JCI116778 7691889 PMC288351

[B129] Mayer M. (2000). Association of serum bilirubin concentration with risk of coronary artery disease. Clin. Chem. 46, 1723–1727 11067805

[B130] McLeod C. Fleeman N. Kirkham J. Bagust A. Boland A. Chu P. (2009). Deferasirox for the treatment of iron overload associated with regular blood transfusions (transfusional haemosiderosis) in patients suffering with chronic anaemia: a systematic review and economic evaluation. Health Technol. Assess. 13, iii–iv, ix–xi, 1–121. 10.3310/hta13010 19068191

[B131] Melamed-Frank M. Lache O. Enav B. I. Szafranek T. Levy N. S. Ricklis R. M. (2001). Structure-function analysis of the antioxidant properties of haptoglobin. Blood 98, 3693–3698 10.1182/blood.V98.13.3693 11739174

[B132] Menke A. Fernandez-Real J. M. Muntner P. Guallar E. (2009). The association of biomarkers of iron status with peripheral arterial disease in US adults. BMC Cardiovasc. Disord. 9:34 10.1186/1471-2261-9-34 19650928 PMC2733106

[B133] Meyers D. G. Jensen K. C. Menitove J. E. (2002). A historical cohort study of the effect of lowering body iron through blood donation on incident cardiac events. Transfusion 42, 1135–1139 10.1046/j.1537-2995.2002.00186.x 12430669

[B134] Meyers D. G. Strickland D. Maloley P. A. Seburg J. K. Wilson J. E. Mcmanus B. F. (1997). Possible association of a reduction in cardiovascular events with blood donation. Heart 78, 188–193 9326996 10.1136/hrt.78.2.188 PMC484902

[B135] Michel J. B. Virmani R. Arbustini E. Pasterkamp G. (2011). Intraplaque haemorrhages as the trigger of plaque vulnerability. Eur. Heart J. 32, 1977–1985, 1985a, 1985b, 1985c. 10.1093/eurheartj/ehr054 21398643 PMC3155759

[B136] Miller D. M. Grover T. A. Nayini N. Aust S. D. (1993). Xanthine oxidase- and iron-dependent lipid peroxidation. Arch. Biochem. Biophys. 301, 1–7 10.1006/abbi.1993.1107 8382902

[B137] Miller M. Hutchins G. M. (1994). Hemochromatosis, multiorgan hemosiderosis, and coronary artery disease. JAMA 272, 231–233 10.1001/jama.1994.03520030073031 8022042

[B138] Miller Y. I. Smith A. Morgan W. T. Shaklai N. (1996). Role of hemopexin in protection of low-density lipoprotein against hemoglobin-induced oxidation. Biochemistry 35, 13112–13117 10.1021/bi960737u 8855948

[B139] Minqin R. Rajendran R. Pan N. Tan B. K. Ong W. Y. Watt F. (2005). The iron chelator desferrioxamine inhibits atherosclerotic lesion development and decreases lesion iron concentrations in the cholesterol-fed rabbit. Free Radic. Biol. Med. 38, 1206–1211 10.1016/j.freeradbiomed.2005.01.008 15808418

[B140] Moran C. J. Siegel M. J. Debaun M. R. (1998). Sickle cell disease: imaging of cerebrovascular complications. Radiology 206, 311–321 9457180 10.1148/radiology.206.2.9457180

[B141] Moreno P. R. Purushothaman K. R. Fuster V. Echeverri D. Truszczynska H. Sharma S. K. (2004). Plaque neovascularization is increased in ruptured atherosclerotic lesions of human aorta: implications for plaque vulnerability. Circulation 110, 2032–2038 10.1161/01.CIR.0000143233.87854.23 15451780

[B142] Morita T. Mitsialis S. A. Koike H. Liu Y. Kourembanas S. (1997). Carbon monoxide controls the proliferation of hypoxic vascular smooth muscle cells. J. Biol. Chem. 272, 32804–32809 10.1074/jbc.272.52.32804 9407056

[B143] Morrison H. I. Semenciw R. M. Mao Y. Wigle D. T. (1994). Serum iron and risk of fatal acute myocardial infarction. Epidemiology 5, 243–246 10.1097/00001648-199403000-00015 8173000

[B144] Mosser D. M. Edwards J. P. (2008). Exploring the full spectrum of macrophage activation. Nat. Rev. Immunol. 8, 958–969 10.1038/nri2448 19029990 PMC2724991

[B145] Mueller P. P. May T. Perz A. Hauser H. Peuster M. (2006). Control of smooth muscle cell proliferation by ferrous iron. Biomaterials 27, 2193–2200 10.1016/j.biomaterials.2005.10.042 16310850

[B146] Munoz-Bravo C. Gutierrez-Bedmar M. Gomez-Aracena J. Garcia-Rodriguez A. Navajas J. F. (2013). Iron: protector or risk factor for cardiovascular disease? Still controversial. Nutrients 5, 2384–2404 10.3390/nu5072384 23857219 PMC3738979

[B147] Nagy E. Eaton J. W. Jeney V. Soares M. P. Varga Z. Galajda Z. (2010). Red cells, hemoglobin, heme, iron, and atherogenesis. Arterioscler. Thromb. Vasc. Biol. 30, 1347–1353 10.1161/ATVBAHA.110.206433 20378845 PMC2893144

[B148] Nagy E. Jeney V. Yachie A. Szabo R. P. Wagner O. Vercellotti G. M. (2005). Oxidation of hemoglobin by lipid hydroperoxide associated with low-density lipoprotein (LDL) and increased cytotoxic effect by LDL oxidation in heme oxygenase-1 (HO-1) deficiency. Cell. Mol. Biol. 51, 377–385 16309588

[B149] Orr A. W. Hastings N. E. Blackman B. R. Wamhoff B. R. (2010). Complex regulation and function of the inflammatory smooth muscle cell phenotype in atherosclerosis. J. Vasc. Res. 47, 168–180 10.1159/000250095 19851078 PMC2842170

[B150] Pang J. H. Jiang M. J. Chen Y. L. Wang F. W. Wang D. L. Chu S. H. (1996). Increased ferritin gene expression in atherosclerotic lesions. J. Clin. Invest. 97, 2204–2212 10.1172/JCI118661 8636399 PMC507299

[B151] Parfenova H. Leffler C. W. Basuroy S. Liu J. Fedinec A. L. (2012). Antioxidant roles of heme oxygenase, carbon monoxide, and bilirubin in cerebral circulation during seizures. J. Cereb. Blood Flow Metab. 32, 1024–1034 10.1038/jcbfm.2012.13 22354150 PMC3367218

[B152] Pasterkamp G. Van Keulen J. K. De Kleijn D. P. (2004). Role of Toll-like receptor 4 in the initiation and progression of atherosclerotic disease. Eur. J. Clin. Invest. 34, 328–334 10.1111/j.1365-2362.2004.01338.x 15147329

[B153] Peyton K. J. Reyna S. V. Chapman G. B. Ensenat D. Liu X. M. Wang H. (2002). Heme oxygenase-1-derived carbon monoxide is an autocrine inhibitor of vascular smooth muscle cell growth. Blood 99, 4443–4448 10.1182/blood.V99.12.4443 12036874

[B154] Philippidis P. Mason J. C. Evans B. J. Nadra I. Taylor K. M. Haskard D. O. (2004). Hemoglobin scavenger receptor CD163 mediates interleukin-10 release and heme oxygenase-1 synthesis: antiinflammatory monocyte-macrophage responses *in vitro*, in resolving skin blisters *in vivo*, and after cardiopulmonary bypass surgery. Circ. Res. 94, 119–126 10.1161/01.RES.0000109414.78907.F9 14656926

[B155] Platt O. S. Brambilla D. J. Rosse W. F. Milner P. F. Castro O. Steinberg M. H. (1994). Mortality in sickle cell disease. Life expectancy and risk factors for early death. N. Engl. J. Med. 330, 1639–1644 10.1056/NEJM199406093302303 7993409

[B156] Pober J. S. Sessa W. C. (2007). Evolving functions of endothelial cells in inflammation. Nat. Rev. Immunol. 7, 803–815 10.1038/nri2171 17893694

[B157] Pocsi I. Jeney V. Kertai P. Emri T. Gyemant G. Fesus L. (2008). Fungal siderophores function as protective agents of LDL oxidation and are promising anti-atherosclerotic metabolites in functional food. Mol. Nutr. Food Res. 52, 1434–1447 10.1002/mnfr.200700467 18646004

[B157a] Porreca E. Ucchino S. Di Febbo C. Di Bartolomeo N. Angelucci D. Napolitano A. M. (1994). Antiproliferative effect of desferrioxamine on vascular smooth muscle cells *in vitro* and *in vivo*. Arterioscler Thromb. 14, 299–304 10.1161/01.ATV.14.2.299 8305423

[B158] Poss K. D. Tonegawa S. (1997). Heme oxygenase 1 is required for mammalian iron reutilization. Proc. Natl. Acad. Sci. U.S.A. 94, 10919–10924 10.1073/pnas.94.20.10919 9380735 PMC23531

[B159] Potor L. Banyai E. Becs G. Soares M. P. Balla G. Balla J. (2013). Atherogenesis may involve the prooxidant and proinflammatory effects of ferryl hemoglobin. Oxid. Med. Cell. Longev. 2013:676425 10.1155/2013/676425 23766856 PMC3671302

[B160] Purushothaman K. R. Purushothaman M. Levy A. P. Lento P. A. Evrard S. Kovacic J. C. (2012). Increased expression of oxidation-specific epitopes and apoptosis are associated with haptoglobin genotype: possible implications for plaque progression in human atherosclerosis. J. Am. Coll. Cardiol. 60, 112–119 10.1016/j.jacc.2012.04.011 22766337

[B161] Radhakrishnan N. Yadav S. P. Sachdeva A. Pruthi P. K. Sawhney S. Piplani T. (2011). Human heme oxygenase-1 deficiency presenting with hemolysis, nephritis, and asplenia. J. Pediatr. Hematol. Oncol. 33, 74–78 10.1097/MPH.0b013e3181fd2aae 21088618

[B162] Rajapurkar M. M. Shah S. V. Lele S. S. Hegde U. N. Lensing S. Y. Gohel K. (2011). Association of catalytic iron with cardiovascular disease. Am. J. Cardiol. 109, 438–442 10.1016/j.amjcard.2011.09.032 22071209

[B163] Rajendran R. Minqin R. Ronald J. A. Rutt B. K. Halliwell B. Watt F. (2012). Does iron inhibit calcification during atherosclerosis? Free Radic. Biol. Med. 53, 1675–1679 10.1016/j.freeradbiomed.2012.07.014 22940067 PMC4831625

[B164] Ramakrishna G. Rooke T. W. Cooper L. T. (2003). Iron and peripheral arterial disease: revisiting the iron hypothesis in a different light. Vasc. Med. 8, 203–210 10.1191/1358863x03vm493ra 14989563

[B165] Rasmussen M. L. Folsom A. R. Catellier D. J. Tsai M. Y. Garg U. Eckfeldt J. H. (2001). A prospective study of coronary heart disease and the hemochromatosis gene (HFE) C282Y mutation: the Atherosclerosis Risk in Communities (ARIC) study. Atherosclerosis 154, 739–746 10.1016/S0021-9150(00)00623-7 11257277

[B166] Recalcati S. Locati M. Marini A. Santambrogio P. Zaninotto F. De Pizzol M. (2010). Differential regulation of iron homeostasis during human macrophage polarized activation. Eur. J. Immunol. 40, 824–835 10.1002/eji.200939889 20039303

[B167] Roest M. Van Der Schouw Y. T. De Valk B. Marx J. J. Tempelman M. J. De Groot P. G. (1999). Heterozygosity for a hereditary hemochromatosis gene is associated with cardiovascular death in women. Circulation 100, 1268–1273 10.1161/01.CIR.100.12.1268 10491369

[B168] Rooyakkers T. M. Stroes E. S. Kooistra M. P. Van Faassen E. E. Hider R. C. Rabelink T. J. (2002). Ferric saccharate induces oxygen radical stress and endothelial dysfunction *in vivo*. Eur. J. Clin. Invest. 32 Suppl. 1, 9–16 10.1046/j.1365-2362.2002.0320s1009.x 11886426

[B169] Rother R. P. Bell L. Hillmen P. Gladwin M. T. (2005). The clinical sequelae of intravascular hemolysis and extracellular plasma hemoglobin: a novel mechanism of human disease. JAMA 293, 1653–1662 10.1001/jama.293.13.1653 15811985

[B170] Rothman S. M. Fulling K. H. Nelson J. S. (1986). Sickle cell anemia and central nervous system infarction: a neuropathological study. Ann. Neurol. 20, 684–690 10.1002/ana.410200606 3813497

[B171] Sacco R. L. Boden-Albala B. Gan R. Chen X. Kargman D. E. Shea S. (1998). Stroke incidence among white, black, and Hispanic residents of an urban community: the Northern Manhattan Stroke Study. Am. J. Epidemiol. 147, 259–268 10.1093/oxfordjournals.aje.a009445 9482500

[B172] Sachais B. S. Turrentine T. Dawicki Mckenna J. M. Rux A. H. Rader D. Kowalska M. A. (2007). Elimination of platelet factor 4 (PF4) from platelets reduces atherosclerosis in C57Bl/6 and apoE-/- mice. Thromb. Haemost. 98, 1108–1113 10.1160/TH07-04-0271 18000617

[B173] Saeed O. Otsuka F. Polavarapu R. Karmali V. Weiss D. Davis T. (2012). Pharmacological suppression of hepcidin increases macrophage cholesterol efflux and reduces foam cell formation and atherosclerosis. Arterioscler. Thromb. Vasc. Biol. 32, 299–307 10.1161/ATVBAHA.111.240101 22095982 PMC3262074

[B174] Sakakura K. Nakano M. Otsuka F. Ladich E. Kolodgie F. D. Virmani R. (2013). Pathophysiology of atherosclerosis plaque progression. Heart Lung Circ. 22, 399–411 10.1016/j.hlc.2013.03.001 23541627

[B175] Sallam T. Cheng H. Demer L. L. Tintut Y. (2013). Regulatory circuits controlling vascular cell calcification. Cell. Mol. Life Sci. 70, 3187–3197 10.1007/s00018-012-1231-y 23269436 PMC3638052

[B176] Salonen J. T. Nyyssonen K. Korpela H. Tuomilehto J. Seppanen R. Salonen R. (1992). High stored iron levels are associated with excess risk of myocardial infarction in eastern Finnish men. Circulation 86, 803–811 10.1161/01.CIR.86.3.803 1516192

[B177] Salonen J. T. Tuomainen T. P. Salonen R. Lakka T. A. Nyyssonen K. (1998). Donation of blood is associated with reduced risk of myocardial infarction. The Kuopio ischaemic heart disease risk factor study. Am. J. Epidemiol. 148, 445–451 10.1093/oxfordjournals.aje.a009669 9737556

[B178] Schwartz S. M. Galis Z. S. Rosenfeld M. E. Falk E. (2007). Plaque rupture in humans and mice. Arterioscler. Thromb. Vasc. Biol. 27, 705–713 10.1161/01.ATV.0000261709.34878.20 17332493

[B179] Schwertner H. A. Jackson W. G. Tolan G. (1994). Association of low serum concentration of bilirubin with increased risk of coronary artery disease. Clin. Chem. 40, 18–23 8287538

[B180] Shen Y. Ward N. C. Hodgson J. M. Puddey I. B. Wang Y. Zhang D. (2013). Dietary quercetin attenuates oxidant-induced endothelial dysfunction and atherosclerosis in apolipoprotein E knockout mice fed a high-fat diet: a critical role for heme oxygenase-1. Free Radic. Biol. Med. 65, 908–915 10.1016/j.freeradbiomed.2013.08.185 24017971

[B181] Silva G. Jeney V. Chora A. Larsen R. Balla J. Soares M. P. (2009). Oxidized hemoglobin is an endogenous proinflammatory agonist that targets vascular endothelial cells. J. Biol. Chem. 284, 29582–29595 10.1074/jbc.M109.045344 19700768 PMC2785591

[B182] Silva M. Silva M. E. De Paula H. Carneiro C. M. Pedrosa M. L. (2008). Iron overload alters glucose homeostasis, causes liver steatosis, and increases serum triacylglycerols in rats. Nutr. Res. 28, 391–398 10.1016/j.nutres.2008.02.009 19083437

[B183] Sindrilaru A. Peters T. Wieschalka S. Baican C. Baican A. Peter H. (2011). An unrestrained proinflammatory M1 macrophage population induced by iron impairs wound healing in humans and mice. J. Clin. Invest. 121, 985–997 10.1172/JCI44490 21317534 PMC3049372

[B184] Singleton J. W. Laster L. (1965). Biliverdin reductase of guinea pig liver. J. Biol. Chem. 240, 4780–4789 4378982

[B185] Siow R. C. Sato H. Mann G. E. (1999). Heme oxygenase-carbon monoxide signalling pathway in atherosclerosis: anti-atherogenic actions of bilirubin and carbon monoxide? Cardiovasc. Res. 41, 385–394 10.1016/S0008-6363(98)00278-8 10341838

[B186] Smith C. Mitchinson M. J. Aruoma O. I. Halliwell B. (1992). Stimulation of lipid peroxidation and hydroxyl-radical generation by the contents of human atherosclerotic lesions. Biochem. J. 286(pt 3), 901–905 1329721 10.1042/bj2860901 PMC1132988

[B187] Smith J. D. Trogan E. Ginsberg M. Grigaux C. Tian J. Miyata M. (1995). Decreased atherosclerosis in mice deficient in both macrophage colony-stimulating factor (op) and apolipoprotein E. Proc. Natl. Acad. Sci. U.S.A. 92, 8264–8268 10.1073/pnas.92.18.8264 7667279 PMC41137

[B188] Solanas-Barca M. Mateo-Gallego R. Calmarza P. Jarauta E. Bea A. M. Cenarro A. (2009). Mutations in HFE causing hemochromatosis are associated with primary hypertriglyceridemia. J. Clin. Endocrinol. Metab. 94, 4391–4397 10.1210/jc.2009-0814 19820015

[B189] Sripetchwandee J. Pipatpiboon N. Chattipakorn N. Chattipakorn S. (2014). Combined therapy of iron chelator and antioxidant completely restores brain dysfunction induced by iron toxicity. PLoS ONE 9:e85115 10.1371/journal.pone.0085115 24400127 PMC3882264

[B190] Stadler N. Lindner R. A. Davies M. J. (2004). Direct detection and quantification of transition metal ions in human atherosclerotic plaques: evidence for the presence of elevated levels of iron and copper. Arterioscler. Thromb. Vasc. Biol. 24, 949–954 10.1161/01.ATV.0000124892.90999.cb 15001454

[B191] Stoneman V. Braganza D. Figg N. Mercer J. Lang R. Goddard M. (2007). Monocyte/macrophage suppression in CD11b diphtheria toxin receptor transgenic mice differentially affects atherogenesis and established plaques. Circ. Res. 100, 884–893 10.1161/01.RES.0000260802.75766.00 17322176 PMC2040259

[B192] Sullivan J. L. (1981). Iron and the sex difference in heart disease risk. Lancet 1, 1293–1294 10.1016/S0140-6736(81)92463-6 6112609

[B193] Sullivan J. L. (1989). The iron paradigm of ischemic heart disease. Am. Heart J. 117, 1177–1188 10.1016/0002-8703(89)90887-9 2653014

[B194] Sullivan J. L. (1991). Blood donation may be good for the donor. Iron, heart disease, and donor recruitment. Vox Sang. 61, 161–164 10.1111/j.1423-0410.1991.tb00940.x 1807057

[B195] Sullivan J. L. (2009). Iron in arterial plaque: modifiable risk factor for atherosclerosis. Biochim. Biophys. Acta 1790, 718–723 10.1016/j.bbagen.2008.06.005 18619522

[B196] Sullivan J. L. Katz S. D. (2007). Iron reduction and cardiovascular outcomes. JAMA 297, 2075–2076 10.1001/jama.297.19.2075-a 17507339

[B197] Sullivan J. L. Zacharski L. R. (2001). Hereditary haemochromatosis and the hypothesis that iron depletion protects against ischemic heart disease. Eur. J. Clin. Invest. 31, 375–377 10.1046/j.1365-2362.2001.00830.x 11380586

[B198] Sun L. Franco O. H. Hu F. B. Cai L. Yu Z. Li H. (2008). Ferritin concentrations, metabolic syndrome, and type 2 diabetes in middle-aged and elderly chinese. J. Clin. Endocrinol. Metab. 93, 4690–4696 10.1210/jc.2008-1159 18796516

[B199] Switzer J. A. Hess D. C. Nichols F. T. Adams R. J. (2006). Pathophysiology and treatment of stroke in sickle-cell disease: present and future. Lancet Neurol. 5, 501–512 10.1016/S1474-4422(06)70469-0 16713922

[B200] Syrovatka P. Kraml P. Hulikova K. Fialova L. Vejrazka M. Crkovska J. (2011). Iron stores are associated with asymptomatic atherosclerosis in healthy men of primary prevention. Eur. J. Clin. Invest. 41, 846–853 10.1111/j.1365-2362.2011.02474.x 21281279

[B201] Tantawy A. A. Adly A. A. El Maaty M. G. Amin S. A. (2009). Subclinical atherosclerosis in young beta-thalassemia major patients. Hemoglobin 33, 463–474 10.3109/03630260903343616 19958191

[B202] Tenhunen R. Marver H. S. Schmid R. (1968). The enzymatic conversion of heme to bilirubin by microsomal heme oxygenase. Proc. Natl. Acad. Sci. U.S.A. 61, 748–755 10.1073/pnas.61.2.748 4386763 PMC225223

[B203] Tolosano E. Fagoonee S. Morello N. Vinchi F. Fiorito V. (2010). Heme scavenging and the other facets of hemopexin. Antioxid. Redox Signal. 12, 305–320 10.1089/ars.2009.2787 19650691

[B204] Tuomainen T. P. Kontula K. Nyyssonen K. Lakka T. A. Helio T. Salonen J. T. (1999). Increased risk of acute myocardial infarction in carriers of the hemochromatosis gene Cys282Tyr mutation: a prospective cohort study in men in eastern Finland. Circulation 100, 1274–1279 10.1161/01.CIR.100.12.1274 10491370

[B205] Tuomainen T. P. Punnonen K. Nyyssonen K. Salonen J. T. (1998). Association between body iron stores and the risk of acute myocardial infarction in men. Circulation 97, 1461–1466 10.1161/01.CIR.97.15.1461 9576426

[B206] Tuomainen T. P. Salonen R. Nyyssonen K. Salonen J. T. (1997). Cohort study of relation between donating blood and risk of myocardial infarction in 2682 men in eastern Finland. BMJ 314, 793–794 10.1136/bmj.314.7083.793 9080998 PMC2126176

[B207] Valenti L. Dongiovanni P. Motta B. M. Swinkels D. W. Bonara P. Rametta R. (2011a). Serum hepcidin and macrophage iron correlate with MCP-1 release and vascular damage in patients with metabolic syndrome alterations. Arterioscler. Thromb. Vasc. Biol. 31, 683–690 10.1161/ATVBAHA.110.214858 21183736

[B208] Valenti L. Swinkels D. W. Burdick L. Dongiovanni P. Tjalsma H. Motta B. M. (2011b). Serum ferritin levels are associated with vascular damage in patients with nonalcoholic fatty liver disease. Nutr. Metab. Cardiovasc. Dis. 21, 568–575 10.1016/j.numecd.2010.01.003 20392616

[B209] Van Tits L. J. Jacobs E. M. Swinkels D. W. Lemmers H. L. Van Der Vleuten G. M. De Graaf J. (2007). Non-transferrin-bound iron is associated with plasma level of soluble intercellular adhesion molecule-1 but not with *in vivo* low-density lipoprotein oxidation. Atherosclerosis 194, 272–278 10.1016/j.atherosclerosis.2006.08.012 16963052

[B210] Vichinsky E. P. (2005). Changing patterns of thalassemia worldwide. Ann. N.Y. Acad. Sci. 1054, 18–24 10.1196/annals.1345.003 16339647

[B211] Vinchi F. De Franceschi L. Ghigo A. Townes T. Cimino J. Silengo L. (2013). Hemopexin therapy improves cardiovascular function by preventing heme-induced endothelial toxicity in mouse models of hemolytic diseases. Circulation 127, 1317–1329 10.1161/CIRCULATIONAHA.112.130179 23446829

[B212] Vinchi F. Gastaldi S. Silengo L. Altruda F. Tolosano E. (2008). Hemopexin prevents endothelial damage and liver congestion in a mouse model of heme overload. Am. J. Pathol. 173, 289–299 10.2353/ajpath.2008.071130 18556779 PMC2438305

[B213] Vinchi F. Tolosano E. (2013). Therapeutic approaches to limit hemolysis-driven endothelial dysfunction: scavenging free heme to preserve vasculature homeostasis. Oxid. Med. Cell. Longev. 2013:396527 10.1155/2013/396527 23781294 PMC3678425

[B214] Virmani R. Kolodgie F. D. Burke A. P. Finn A. V. Gold H. K. Tulenko T. N. (2005). Atherosclerotic plaque progression and vulnerability to rupture: angiogenesis as a source of intraplaque hemorrhage. Arterioscler. Thromb. Vasc. Biol. 25, 2054–2061 10.1161/01.ATV.0000178991.71605.18 16037567

[B215] Vukovic I. Arsenijevic N. Lackovic V. Todorovic V. (2006). The origin and differentiation potential of smooth muscle cells in coronary atherosclerosis. Exp. Clin. Cardiol. 11, 123–128 18651048 PMC2274860

[B216] Wang H. Luo W. Wang J. Guo C. Wolffe S. L. Sun E. B. (2013). Paradoxical protection from atherosclerosis and thrombosis in a mouse model of sickle cell disease. Br. J. Haematol. 162, 120–129 10.1111/bjh.12342 23590132 PMC4780334

[B217] Watari Y. Yamamoto Y. Brydun A. Ishida T. Mito S. Yoshizumi M. (2008). Ablation of the bach1 gene leads to the suppression of atherosclerosis in bach1 and apolipoprotein E double knockout mice. Hypertens. Res. 31, 783–792 10.1291/hypres.31.783 18633191

[B217a] Wong C. M. Preston I. R. Hill N. S. Suzuki Y. J. (2012). Iron chelation inhibits the development of pulmonary vascular remodeling. Free Radic. Biol. Med. 53, 1738–1747 10.1016/j.freeradbiomed.2012.08.576 22974762 PMC3472156

[B218] Wu B. J. Kathir K. Witting P. K. Beck K. Choy K. Li C. (2006). Antioxidants protect from atherosclerosis by a heme oxygenase-1 pathway that is independent of free radical scavenging. J. Exp. Med. 203, 1117–1127 10.1084/jem.20052321 16606673 PMC2118288

[B219] Yachie A. Niida Y. Wada T. Igarashi N. Kaneda H. Toma T. (1999). Oxidative stress causes enhanced endothelial cell injury in human heme oxygenase-1 deficiency. J. Clin. Invest. 103, 129–135 10.1172/JCI4165 9884342 PMC407858

[B220] Yet S. F. Layne M. D. Liu X. Chen Y. H. Ith B. Sibinga N. E. (2003). Absence of heme oxygenase-1 exacerbates atherosclerotic lesion formation and vascular remodeling. FASEB J. 17, 1759–1761 10.1096/fj.03-0187fje 12958201

[B221] You S. A. Archacki S. R. Angheloiu G. Moravec C. S. Rao S. Kinter M. (2003). Proteomic approach to coronary atherosclerosis shows ferritin light chain as a significant marker: evidence consistent with iron hypothesis in atherosclerosis. Physiol. Genomics 13, 25–30 10.1152/physiolgenomics.00124.2002 12644631

[B222] Yuan X. M. Anders W. L. Olsson A. G. Brunk U. T. (1996). Iron in human atheroma and LDL oxidation by macrophages following erythrophagocytosis. Atherosclerosis 124, 61–73 10.1016/0021-9150(96)05817-0 8800494

[B223] Zacharski L. R. Chow B. K. Howes P. S. Shamayeva G. Baron J. A. Dalman R. L. (2007). Reduction of iron stores and cardiovascular outcomes in patients with peripheral arterial disease: a randomized controlled trial. JAMA 297, 603–610 10.1001/jama.297.6.603 17299195

[B224] Zacharski L. R. Ornstein D. L. Woloshin S. Schwartz L. M. (2000). Association of age, sex, and race with body iron stores in adults: analysis of NHANES III data. Am. Heart J. 140, 98–104 10.1067/mhj.2000.106646 10874269

[B225] Zarjou A. Jeney V. Arosio P. Poli M. Antal-Szalmas P. Agarwal A. (2009). Ferritin prevents calcification and osteoblastic differentiation of vascular smooth muscle cells. J. Am. Soc. Nephrol. 20, 1254–1263 10.1681/ASN.2008070788 19423691 PMC2689905

[B226] Zenke-Kawasaki Y. Dohi Y. Katoh Y. Ikura T. Ikura M. Asahara T. (2007). Heme induces ubiquitination and degradation of the transcription factor Bach1. Mol. Cell. Biol. 27, 6962–6971 10.1128/MCB.02415-06 17682061 PMC2099246

[B227] Zhang W. J. Frei B. (2003). Intracellular metal ion chelators inhibit TNFalpha-induced SP-1 activation and adhesion molecule expression in human aortic endothelial cells. Free Radic. Biol. Med. 34, 674–682 10.1016/S0891-5849(02)01375-8 12633744

[B228] Zhang W. J. Wei H. Frei B. (2010). The iron chelator, desferrioxamine, reduces inflammation and atherosclerotic lesion development in experimental mice. Exp. Biol. Med. 235, 633–641 10.1258/ebm.2009.009229 20463304 PMC3057189

[B229] Zheng H. Cable R. Spencer B. Votto N. Katz S. D. (2005). Iron stores and vascular function in voluntary blood donors. Arterioscler. Thromb. Vasc. Biol. 25, 1577–1583 10.1161/01.ATV.0000174126.28201.61 15961703

[B230] Zohn I. E. De Domenico I. Pollock A. Ward D. M. Goodman J. F. Liang X. (2007). The flatiron mutation in mouse ferroportin acts as a dominant negative to cause ferroportin disease. Blood 109, 4174–4180 10.1182/blood-2007-01-066068 17289807 PMC1885502

